# Cord blood metabolites and rapid postnatal growth as multiple mediators in the prenatal propensity to childhood overweight

**DOI:** 10.1038/s41366-022-01108-0

**Published:** 2022-05-04

**Authors:** Rossella Alfano, Michelle Plusquin, Oliver Robinson, Sonia Brescianini, Lida Chatzi, Pekka Keski-Rahkonen, Evangelos Handakas, Lea Maitre, Tim Nawrot, Nivonirina Robinot, Theano Roumeliotaki, Franco Sassi, Augustin Scalbert, Martine Vrijheid, Paolo Vineis, Lorenzo Richiardi, Daniela Zugna

**Affiliations:** 1 Centre for Environmental Sciences, Hasselt University, Diepenbeek, Belgium; 2 Μedical Research Council-Health Protection Agency Centre for Environment and Health, Imperial College London, London, UK; 3 Centre for Behavioural Science and Mental Health, Istituto Superiore di Sanità, Rome, Italy; 4 Department of Preventive Medicine, Keck School of Medicine, University of Southern California, Los Angeles, USA; 5 Nutrition and Metabolism Branch, International Agency for Research on Cancer, Lyon, France; 6 Barcelona Institute of Global Health (ISGlobal), Barcelona, Spain; 7 Department of Social Medicine, Faculty of Medicine, University of Crete, Heraklion, Greece; 8 Centre for Health Economics & Policy Innovation, Department of Economics & Public Policy, Imperial College Business School, South Kensington Campus, London, UK; 9 Universitat Pompeu Fabra (UPF), Barcelona, Spain; 10 CIBER Epidemiología y Salud Pública (CIBERESP), Madrid, Spain; 11 Cancer Epidemiology Unit, Department of Medical Sciences, University of Turin and CPO‐Piemonte, Torino, Italy

**Keywords:** Risk factors, Epidemiology

## Abstract

**Background:**

The mechanisms underlying childhood overweight and obesity are poorly known. Here, we investigated the direct and indirect effects of different prenatal exposures on offspring rapid postnatal growth and overweight in childhood, mediated through cord blood metabolites. Additionally, rapid postnatal growth was considered a potential mediator on childhood overweight, alone and sequentially to each metabolite.

**Methods:**

Within four European birth-cohorts (*N* = 375 mother-child dyads), information on seven prenatal exposures (maternal education, pre-pregnancy BMI, weight gain and tobacco smoke during pregnancy, age at delivery, parity, and child gestational age), selected as obesogenic according to a-priori knowledge, was collected. Cord blood levels of 31 metabolites, associated with rapid postnatal growth and/or childhood overweight in a previous study, were measured via liquid-chromatography-quadrupole-time-of-flight-mass-spectrometry. Rapid growth at 12 months and childhood overweight (including obesity) between four and eight years were defined with reference to WHO growth charts. Single mediation analysis was performed using the imputation approach and multiple mediation analysis using the extended-imputation approach.

**Results:**

Single mediation suggested that the effect of maternal education, pregnancy weight gain, parity, and gestational age on rapid postnatal growth but not on childhood overweight was partly mediated by seven metabolites, including cholestenone, decenoylcarnitine(C10:1), phosphatidylcholine(C34:3), progesterone and three unidentified metabolites; and the effect of gestational age on childhood overweight was mainly mediated by rapid postnatal growth. Multiple mediation suggested that the effect of gestational age on childhood overweight was mainly mediated by rapid postnatal growth and that the mediating role of the metabolites was marginal.

**Conclusion:**

Our findings provide evidence of the involvement of in utero metabolism in the propensity to rapid postnatal growth and of rapid postnatal growth in the propensity to childhood overweight. We did not find evidence supporting a mediating role of the studied metabolites alone between the studied prenatal exposures and the propensity to childhood overweight.

## Introduction

Over the last decades, childhood obesity prevalence has risen globally to the level of an epidemic [1]. Because of its immediate and delayed health consequences [2, 3] related to its persistence later in life [4], childhood obesity is a major public health problem. Although energy imbalance between food intake and physical activity expenditure is the most obvious determinant of childhood obesity, underlying causes are complex, include the interplay between genetic and non genetic factors, and may originate already during prenatal development [5, 6]. According to the thrifty phenotype hypothesis, exposure to detrimental prenatal factors can induce permanent changes in foetus metabolism that promote storage of excess calories, predisposing children to weight gain and leading to obesity in childhood [7]. Previous epidemiological studies reported an increased risk of obesity in childhood in association with adverse prenatal factors including high pre-pregnancy maternal body mass index (BMI), high maternal weight gain during the pregnancy, socioeconomic disadvantage, smoking during pregnancy, nulliparity, younger maternal age and shorter gestation [8–15]. However, the potential pathways underlying these associations are poorly understood.

Prenatal exposures may affect the metabolome of the child [16, 17], and previously we found some cord blood metabolites at birth to be associated with obesity later in childhood and rapid postnatal growth during the first years of life [18]. Taken together, these studies suggest a causal link between prenatal exposures and child obesity, potentially involving altered metabolism *in utero* and rapid postnatal growth [19]. The role of these causal paths has been little investigated.

**Fig. 1:**
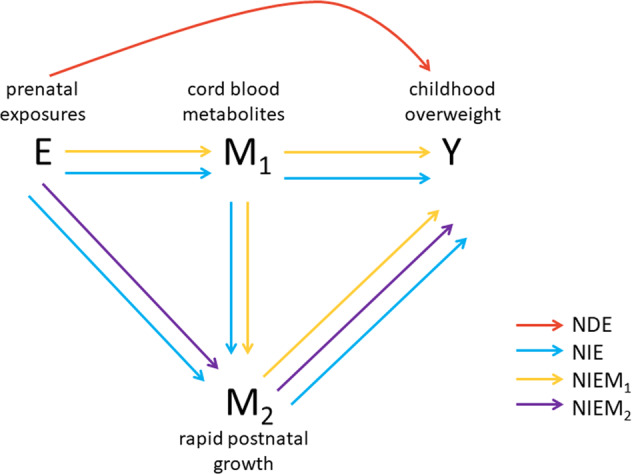
Directed acylic graph of assumed causal relationships. The simplified directed acyclic graph displays the hypothetical causal relationships linking prenatal exposure (E), cord blood metabolites (M_1_), rapid postnatal growth (M_2_) and childhood overweight (Y). NDE: natural direct effect; NIE: natural indirect effect; NIEM_1_: natural indirect effect through M_1_; NIEM_2_: natural indirect effect through M_2_. For simplicity, confounders of the E-Y, E-M1, E-M2, M1-M2, M1-Y, M2-Y associations were not shown.

In this study, we investigated the total and mediated effects of obesogenic prenatal exposures, including maternal education, pre-pregnancy body mass index (BMI), maternal weight gain during the pregnancy, tobacco smoke during pregnancy, maternal age at delivery, gestational age, and parity, on childhood overweight. We decomposed the total effect into a direct and an indirect (mediated) effect through sequential mediation of selected cord blood metabolites at birth and rapid postnatal growth (Fig. 1).

## Materials and methods

### Study population

The study population arises from a subset of children (*N* = 500) from four independent European population-based birth-cohorts: the ENVIRonmental influence ON early AGEing (ENVIR*ON*AGE) cohort (*N* = 200) [20], the INfancia y Medio Ambiente (INMA) cohort (*N* = 100) [21], the Piccolipiù cohort (*N* = 99) [22], and the Rhea cohort (*N* = 101) [23, 24], that participate in the STOP and EXPOsOMICS projects [25]. The study was approved by the ethics committees of the Hasselt University and the Hospital East-Limburg for ENVIR*ON*AGE; of the Hospital del Mar Medical Research Institute for INMA; of the Local Health Unit Roma E (management centre), of the Istituto Superiore di Sanità (National Institute of Public Health) and of each local centre for Piccolipiù; and of the University Hospital at Heraklion for Rhea. Informed consent for participation was provided by parents or mothers. A brief description of individual cohorts and collection of covariates is presented in the Supplementary Methods. Complete case study population (Table S1) included 375 children (104 ENVIRONAGE, 86 INMA, 97 Piccolipiù and 88 Rhea) for the analysis involving the metabolites as mediators of the effect of prenatal exposures on rapid postnatal growth; and 249 children (83 of INMA, 78 of Piccolipiù and 88 of Rhea) for the mediation analyses on childhood overweight.

### Variables definition

#### Prenatal exposures

Prenatal exposures were selected as being associated with childhood obesity according to a-priori knowledge [9–15]. Maternal education level (categorised in low if the mother had no diploma or primary school diploma, medium if the mother had secondary school diploma, and high if the mother had a university degree or higher education qualification), maternal pre-pregnancy BMI (in kilograms over squared metres), maternal weight gain over the pregnancy (in kilograms), maternal tobacco smoke at any time during pregnancy (dichotomised into smoker or non-smoker), maternal age (in years), gestational age (in weeks) and parity (coded as primiparity or multiparity) were collected through questionnaires at enrolment or from medical data files.

Based on the aforementioned prenatal exposures, an obesogenic factor score was built (Table S2). In brief, for each prenatal exposure, a score between 0 and 1 was assigned according to the categorical coding of the exposure (with 0 meaning low risk for obesity and 1 meaning high risk according to a-priori knowledge). The obesogenic factor score resulted from the sum of each prenatal exposure score and ranged between 0 and 7.

#### Cord blood metabolites

Untargeted metabolomics of serum (INMA and Rhea) and plasma (ENVIR*ON*AGE and Piccolipiù) samples were measured from 499 cord blood samples (200 ENVIR*ON*AGE, 100 INMA, 99 Piccolipiù and 100 Rhea) using reversed-phase liquid chromatography-quadrupole time-of-flight mass spectrometry (UHPLC-QTOF-MS) (details of the sample analysis and data preprocessing were described earlier [26]).

Over the total 4,712 metabolic features, we included 32 metabolic features annotated to 31 metabolites, of which 10 were unidentified metabolites (Table S3). These 31 metabolites were selected as they have been previously associated with rapid growth at 12 months and/or childhood overweight [18].

In detail, via a metabolome wide association study (MWAS) four metabolites were associated with rapid growth at 12 months (cholestenone and three unidentified (U) metabolites: U4, U6 and U8) and eight with childhood overweight (valine, and seven U metabolites: U1, U2, U3, U4, U5, U7 and U9) using false discovery rate (FDR) adjusted p values threshold of 0.05 upon adjustment for child sex and age at outcome measurement, cohort and ethnicity [18]. We included in the present study the metabolic feature (N = 12) with the highest intensity annotated to each of the identified metabolites for each outcome. These features were: mass to charge ratio (m/z) 385.3487 annotated as cholestenone, m/z 72.08108 as valine, m/z 129.0025 as U1, m/z 196.9619 as U2, m/z 242.9253 as U3, m/z 169.134 as U5, m/z 289.2157 as U6, m/z 209.1159 as U7, m/z 269.1894 as U8 and m/z 460.4366 as U9. Since two different features of the same metabolite, U4, had the highest intensity in rapid postnatal growth (m/z 482.2392) and childhood overweight analyses (m/z 154.0264), we included both of them in our analysis (Table S3).

We lowered the significance threshold used in the original study [18] to crude p values of 0.05 to seek features associated with both rapid postnatal growth and childhood overweight and identified two additional metabolic features (m/z 175.1079 and m/z 202.0483) (Table S3). Following an identification strategy similar to this previously described by Robinson et al. [26] and reporting the level of identification as proposed by Sumner et al. [27], we found that the feature of m/z 202.0483 is hippuric acid while we could not identify the feature of m/z 175.1079 (Table S3).

Additionally, we included a set of 18 metabolites associated with rapid postnatal growth (indolelactic acid, cholesterol, decenoylcarnitine (C10:1), progesterone, docosahexaenoic acid, tetradecadiencarnitine (C14:2), cholestenone, lysoPC (C20:2), lysoPC (C20:4), PC (C30:0), PC (C32:0), PC (C34:2), plasmalogen PC(C36:4) or PC(O-36:5), plasmalogen PC(C36:3) or PC(O-36:4), PC (C36:4), PC (C36:4) isomer, PC (C38:4), plasmalogen PC(C38:4) or PC(O-38:5)) and two metabolites associated with childhood overweight (leucine and docosahexaenoic acid), in the same study, in look-up analyses of 43 pre-annotated metabolites (using the same model described above for the MWAS and crude p value threshold of 0.05) [18] (Table S3).

#### Rapid postnatal growth

Rapid postnatal growth was defined as the difference between World Health Organization (WHO)-standard deviation (SD)-score of birthweight (obtained from obstetric records) and predicted weight at 12 months >0.67 SD according to Ong et al. [28]. Sex- and age-specific predicted weight at 12 months was calculated via a two-step prediction approach using fractional polynomials of age by gender in each cohort [18]. Since ENVIRONAGE follow-up weight data were collected for only a selection of children (55% of the total population) participating in the two years of age sub-study, rapid growth measurements were available in 396 (109 ENVIR*ON*AGE, 87 INMA, 99 Piccolipiù and 101 Rhea) children.

#### Childhood overweight

Sex- and age-adjusted SD BMI scores during childhood were calculated using WHO growth reference. BMI was calculated as the ratio of child weight (in kilograms) over squared height (in metres) self-reported from parents or measured by trained staff between four and eight years of age, using the closest measurement to six years of age if multiple measures were available [median age (range) = 6.11 (4.04–7.49) years in INMA, 4.41 (4.17–4.74) years in Piccolipiù, 6.00 (4.01–7.06) years in Rhea]. Childhood overweight (including children with obesity) was defined based on child BMI SD score: (i) >2 in children under five years and >1 in children older than five years, according to the WHO cut-offs [29], in the main analyses; and (ii) > age- and sex-specific BMI cut-offs, according to the International Obesity Task Force (IOTF) [30], in sensitivity analyses.

Out of 500 children enroled in the study at birth, 275 (96 INMA, 79 Piccolipiù and 100 Rhea) children had BMI data available between four and eight years of age. Out of 200 participants of the ENVIR*ON*AGE cohort, only seven children had been followed-up after four years of age. Hence, we excluded the ENVIR*ON*AGE cohort from the analysis of childhood overweight.

#### Covariates

The sex of the newborns was collected from the medical data files. Child ethnicity based on maternal or grandparent’s origin was collected through questionnaires at enrolment. Child age at BMI measurement in childhood was reported by parents or trained staff.

### Statistical analysis

Figure 1 conceptualises the casual relationship we investigated in this study linking prenatal exposure (E), cord blood metabolites (M_1_), rapid postnatal growth (M_2_) and childhood overweight (Y).

The total effects (TE), with 95% CI confidence intervals, of each E on M_2_ and on Y were assessed via logistic regressions. All the analyses were adjusted for sex of the newborns, child ethnicity, cohort membership, child age at the measurement of BMI (in analyses with childhood overweight as the outcome), and the other selected obesogenic prenatal exposures (except gestational age and maternal weight gain during the pregnancy that were not included as covariates as they are potentially affected by the other prenatal exposures). Analyses on maternal education were adjusted only for sex, cohort, ethnicity, and child age at the BMI measurement (when childhood overweight was the outcome of interest) since all the other prenatal exposures might lay on the path of the effect of maternal education on rapid postnatal growth/childhood overweight.

The natural indirect effect (NIE), operating via the given mediator, and the natural direct effect (NDE), unexplained by the given mediator were estimated via single and multiple mediation analysis.

First, we performed model-based single mediation analysis using the imputation approach [31] to quantify the effects of: (i) E on M_2_ mediated by M_1_; and E on Y mediated by (ii) M_1_ or (iii) M_2_ as single separated mediators. We decomposed the TE into the NDE and the NIE.

Second, we used the extended-imputation approach [32] to quantify the sequential mediation effect of M_1_ and M_2_ in the association between E and Y. We estimated the TE, the NDE, and the NIE, which was further refined into NIE through M_1_ (NIEM_1_) representing the pathways E-M_1_-Y and E-M_1_-M_2_-Y, and the NIE through only M_2_ (NIEM_2_) representing the pathway E-M_2_-Y.

In all the mediation analyses, the outcome was modelled against each prenatal exposure using a logistic regression model, adjusted for the same confounders described previously. 95% CIs were calculated by bootstrap with 1000 replications. In the text and in the figures, we reported the TE, NIE and NDE as odds ratios (OR). The TE OR expresses the effect of Y on E. The NIE and NDE ORs express the effect of E on Y, mediated and unmediated by M.

Third, we performed sensitivity analyses:

(i) using IOTF cut-offs to define childhood overweight;
(ii) using the first two principal components of the 32 cord blood metabolic features as the mediators instead of the single metabolites (Figure S1);
(iii) excluding preterm children (<37 weeks of gestation) in the analyses of gestational age;
(iv) excluding children born by caesarean delivery.

Fourth, the single mediation analysis of the effect of prenatal exposures on rapid postnatal growth was repeated in the smaller subset of 249 children included in the analyses on childhood overweight.

## Results

Table 1 shows the characteristics of the study populations. The obesogenic score was positively associated with both rapid growth (*p* value = 6.34e−04) and childhood overweight (*p* value = 2.69e−05). Upon adjustment for confounders, children were at higher risk of becoming overweight in childhood if they were rapid growers at 12 months (OR = 4.39 95% CI = 2.14–9.28, Table S4).

**Table 1:** Descriptive characteristics of the study population.

	Study population postnatal (12 months) rapid growth analyses *N* = 375			Study population childhood overweight analyses *N* = 249		
	Normal growth *N* = 268 (71.47%)	Rapid growth *N* = 107 (28.53%)	*P* value	Normal weight *N* = 193 (77.51%)	Overweight *N* = 56 (22.49%)	*P* value
Cohort membership			**<0.01**			**<0.01**
ENVIRONAGE	73 (27.24%)	31 (28.97%)		-	-	
INMA	62 (23.13%)	24 (22.43%)		58 (30.05%)	25 (44.64%)	
Piccolipiù	81 (30.22%)	16 (14.95%)		74 (38.34%)	4 (7.14%)	
Rhea	52 (19.40%)	36 (33.64%)		61 (31.61%)	27 (48.21%)	
Sex, boy	146 (54.48%)	45 (42.06%)	**0.04**	97 (50.26%)	30 (53.57%)	0.78
Child ethnicity, maternal origin different from study country	22 (8.21%)	11 (10.28%)	0.66	12 (6.22%)	2 (3.57%)	0.67
Delivery, caesarean	66 (24.72)	33 (30.84)	0.28	67 (34.90)	24 (42.86)	0.35
Gestation age, weeks	39.54 (±1.36)	38.36 (±1.72)	**<0.01**	39.33 (±1.51)	38.88 (±1.61)	0.05
Gestation age, <37 weeks	9 (3.36%)	16 (14.95%)	**<0.01**	9 (4.66%)	5 (8.93%)	0.37
Parity, primiparous	119 (44.40%)	58 (54.21%)	0.11	76 (39.38%)	28 (50.00%)	0.21
Maternal age, years	31.34 (±4.43)	30.28 (±4.68)	**0.04**	32.17 (±4.62)	30.50 (±4.95)	**0.02**
Maternal age, <32 years	151 (56.34%)	69 (64.49%)	0.18	89 (46.11%)	38 (67.86%)	**<0.01**
Maternal education level,			0.07			0.36
(i) Low (no diploma or primary school diploma)	28 (10.45%)	14 (13.08%)		21 (10.88%)	8 (14.29%)	
(ii) Medium (secondary school diploma)	105 (39.18%)	53 (49.53%)		90 (46.63%)	30 (53.57%)	
(iii) High (university degree or higher education qualification)	135 (50.37%)	40 (37.38%)		82 (42.49%)	18 (32.14%)	
Pre-pregnancy maternal BMI, Kilogram/ metre^2^	23.64 (±4.43)	24.27 (±4.74)	0.22	23.14 (±4.35)	25.73 (±4.72)	**<0.01**
Prepregnancy maternal BMI, Overweight or obese (BMI ≥ 25)	77 (28.73%)	35 (32.71%)	0.52	52 (26.94%)	25 (44.64%)	**0.02**
Maternal weight gain over the pregnancy, Kilograms	13.52 (5.03)	13.63 (5.55)	0.85	12.96 (4.79)	14.20 (5.95)	0.11
Maternal weight gain over the pregnancy, excess	83 (30.97%)	38 (35.51%)	0.47	49 (25.39%)	27 (48.21%)	**<0.01**
Maternal pregnancy tobacco smoke, yes	42 (15.67%)	23 (21.50%)	0.23	35 (18.13%)	14 (25.00%)	0.34
Obesogenic factors score, numeric (range 0-7)	2.10 (±1.30)	2.61 (±1.30)	**<0.01**	1.95 (±1.27)	2.86 (±1.37)	**<0.01**
Postnatal rapid growth at 12 months, yes	0 (0.00%)	107 (100.00%)	**<0.01**	40 (20.73%)	30 (53.57%)	**<0.01**
Child overweight (WHO cut-off), yes	26 (14.21%)	30 (41.67%)	**<0.01**	0 (0.00%)	56 (100.00%)	**<0.01**
Child overweight (IOTF cut-offs), yes	29 (15.85%)	33 (45.83%)	**<0.01**	11 (5.70%)	49 (87.50%)	**<0.01**
Child age at the BMI measurement, years	5.33 (1.00)	5.48 (1.03)	0.30	5.25 (1.00)	5.93 (0.82)	**<0.01**

Number (%) and mean (±standard deviation) are reported for categorical and continuous variables, respectively. *P* values from chi-square and *t*-test statistics are reported for categorical and continuous variables, respectively, and are bolded if <0.05.

### Analysis on rapid growth with cord blood metabolites as single mediators

Upon adjustment for confounders, children were at higher risk of experiencing rapid growth if they were born from primiparous mothers (TE OR of primiparous versus (vs) pluriparous = 1.93, 95% CI = 1.14–1.31) and had higher obesogenic factors score (TE per each unit of the score OR = 1.33 95% CI = 1.11–1.61), and at lower risk, if they were born after a longer gestation (TE OR = 0.56 per each week of gestation, 95% CI = 0.46–0.67) (Table S4). Associations of rapid growth with most of the other prenatal exposures were in the expected direction, including positive association with low education (TE OR of maternal low vs high education = 1.85, 95% CI = 0.83–4.03) and negative association with weight gain during the pregnancy (TE OR = 0.98 per each kilogram of weight, 95% CI = 0.94–1.03) (Table S4).

**Fig. 2:**
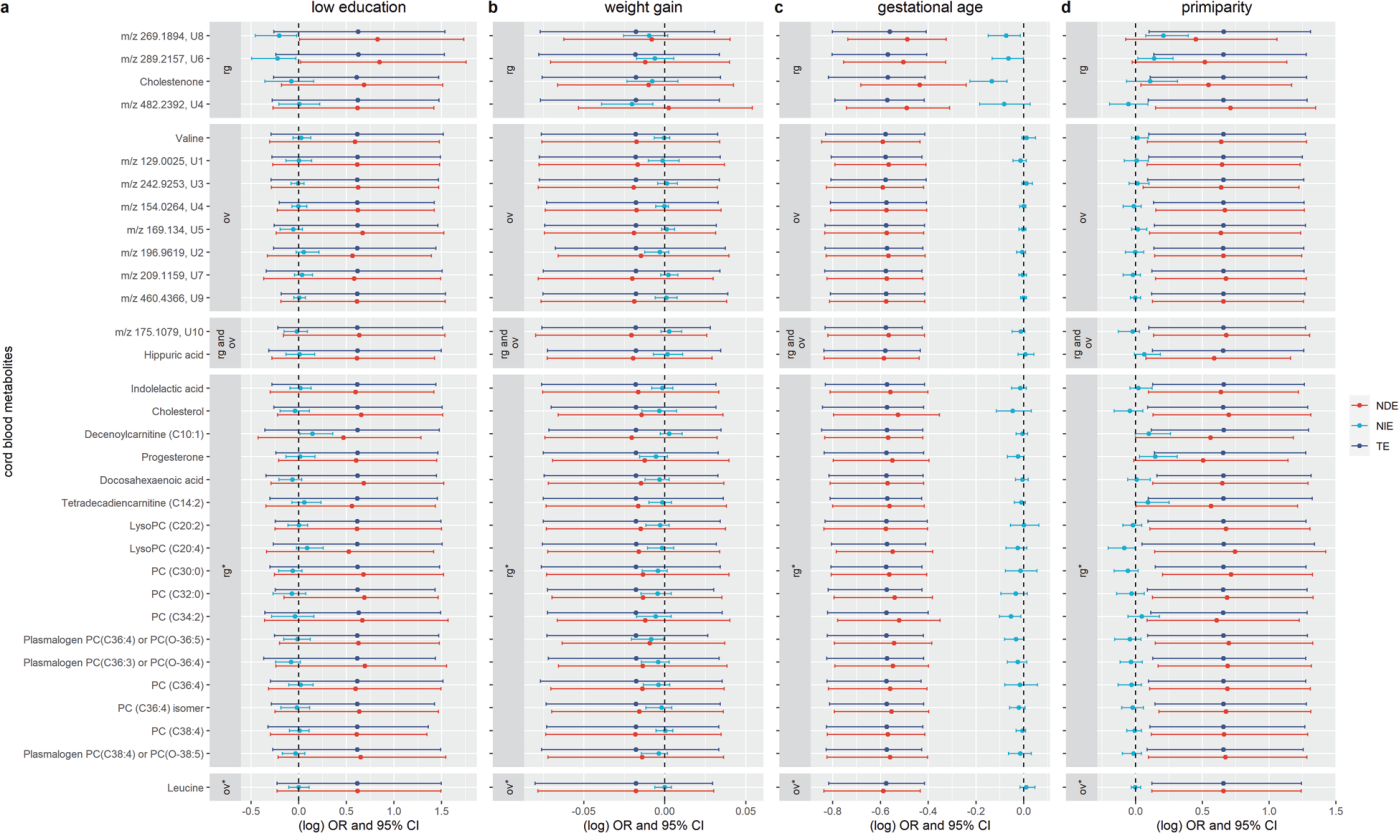
Effects of prenatal exposures on rapid postnatal growth through cord blood metabolites. The plots represent on the x-axis the (log) point estimates odds ratio (dots) and 95% confidence intervals (bars) from single mediation analysis (*N* = 375) for the effects of **a** maternal low vs high education, **b** one kilogram increase of maternal weight during the pregnancy, **c** one week increase of gestation, and **d** primiparous vs pluriparous on the rapid postnatal growth through the metabolites—grouped in five sets: metabolites previously related with rapid growth (rg), overweight (ov), and rapid growth and overweight (rg and ov) in the MWAS, and metabolites previously related with rapid growth (rg*) and overweight (ov*) in the look-up analyses of Handakas et al. [18]. All the analyses are adjusted for sex of the newborns, child ethnicity and cohort membership. The analyses of maternal weight gain, gestational age and parity are additionally adjusted for maternal education, pre-pregnancy BMI, smoking, age at delivery, and parity. CI: confidence intervals, NIE: natural indirect effect; NDE: natural direct effect; OR: odds ratio; TE: total effect.

The effect of maternal education, weight gain during the pregnancy, gestational age and parity, on rapid growth was partly mediated, as represented in the Fig. 2, by the metabolites previously found associated with rapid growth by Handakas et al: [18]. the effect of maternal low vs high education on rapid growth was mediated by metabolite U6 (*m/z* 289.2157, NIE OR of maternal low vs high education = 0.80, 95% CI = 0.62–0.97), metabolite U8 (*m/z* 269.1894, NIE OR of maternal low vs high education = 0.81, 95% CI = 0.65–0.99) and decenoylcarnitine (C10:1) (NIE OR of maternal low vs high education = 1.16, 95% CI = 1.01–1.43) (Fig. 2a); the effect of maternal weight gain during the pregnancy on rapid growth was mediated by metabolite U4 (*m/z* 482.2392, NIE OR per each kilogram of weight = 0.98, 95% CI = 0.96–0.99) (Fig. 2b); the effect of gestational age on rapid growth was mediated by metabolite U8 (*m/z* 269.1894, NIE OR per each week of gestation = 0.93, 95% CI = 0.86–0.99), cholestenone (NIE OR per each week of gestation = 0.87, 95% CI = 0.79–0.94) and phospatidilcholine (PC) (C34:2) (NIE OR per each week of gestation = 0.95, 95% CI = 0.90–0.99) (Fig. 2c); the effect of being primiparous on rapid growth was mediated by metabolite U6 (*m/z* 289.2157, NIE OR of primiparous vs pluriparous = 1.15, 95% CI = 1.02–1.35), metabolite U8 (*m/z* 269.1894, NIE OR of primiparous vs pluriparous = 1.23, 95% CI = 1.07–1.48) and progesterone (NIE OR of primiparous vs pluriparous = 1.16, 95% CI = 1.03–1.37) (Fig. 2d). There was no evidence that the association of other prenatal exposures with rapid growth was mediated by the cord blood metabolites examined (Fig. S2).

Using the subset of children (*N* = 249) participating in the analysis of childhood overweight, the point estimates of the mediation effects remained similar, apart for those of education via U6 and U8 that were strongly attenuated, and of parity via U6 that faded (Fig. S3).

### Analysis on childhood overweight with blood metabolites or rapid growth as single mediators

Upon adjustment for confounders, childhood overweight was associated with higher pre-pregnancy BMI (TE OR per each unit of BMI = 1.10, 95% CI = 1.02–1.18) and obesogenic factors score (TE per each unit of the score OR = 1.61, 95% CI = 1.25–2.11) (Table S4). Associations of childhood overweight with most of the other prenatal exposures were in the expected direction, including negative association with gestational age (TE OR per each week of gestation = 0.80 per each week of gestation, 95% CI = 0.62–1.02), and positive association with low education (TE OR of maternal low vs high education = 1.21, 95% CI = 0.42–3.36) and primiparity (TE OR of primiparous vs pluriparous = 1.90, 95% CI = 0.91–4.01) (Table S4).

**Fig. 3:**
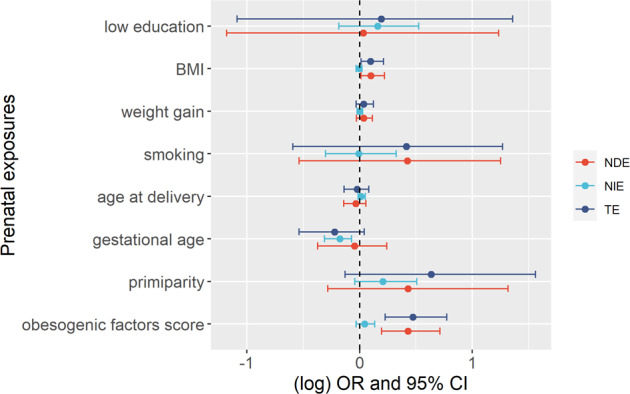
Effects of prenatal exposures on childhood overweight through rapid postnatal growth. The plot represents on the *x*-axis the (log) point estimates odds ratio (dots) and 95% confidence intervals (bars) from single mediation for the effects of the prenatal exposures, listed on the *y*-axis (maternal low vs high education, one unit increase of BMI, one kilogram increase of maternal weight during the pregnancy, smokers vs non-smokers during the pregnancy, one year increase of age at delivery, one week increase of gestation, primiparous vs pluriparous, one unit increase in obesogenic factors score) on childhood overweight through postnatal rapid weight gain. All the analyses are adjusted for sex of the newborns, child ethnicity, cohort membership and child age at the measurement of BMI. The analyses of maternal pre-pregnancy BMI, weight gain, smoking, age at delivery, gestational age and parity are additionally adjusted for maternal education, pre-pregnancy BMI, smoking, age at delivery, and parity. CI: confidence intervals, NIE: natural indirect effect; NDE: natural direct effect; OR: odds ratio, TE: total effect.

There was no evidence that the effect of the prenatal exposure examined on childhood overweight was mediated by the cord blood metabolites under study (Fig. S4). Despite being non significant, the effect of pre-pregnancy maternal BMI, gestational age and the obesogenic factors score on childhood overweight was mediated by valine and leucine, with direction of the mediation effects in the opposite direction to the total effects. Most of the effect of gestational age, of low vs high educational level and part of the effect of being born from a primiparous mother on childhood overweight was mediated by rapid growth (NIE OR per each week of gestation = 0.84, 95% CI = 0.72–0.93; NIE OR of maternal low vs high education = 1.17, 95% CI = 0.83–1.69; NIE OR of primiparous vs pluriparous = 1.23, 95% CI = 0.96–1.66; Fig. 3). The effect of the other four prenatal exposures examined (maternal pre-pregnancy BMI, smoking and weight gain during the pregnancy, and age at delivery) was mainly direct, as summarised in the analysis of the obesogenic factors score, whose effect on childhood overweight was direct (NDE OR per each unit of the score = 1.54, 95% CI = 1.21–2.04) and not mediated by rapid growth (Fig. 3).

### Analysis on childhood overweight with blood metabolites and rapid growth as multiple mediators

**Fig. 4:**
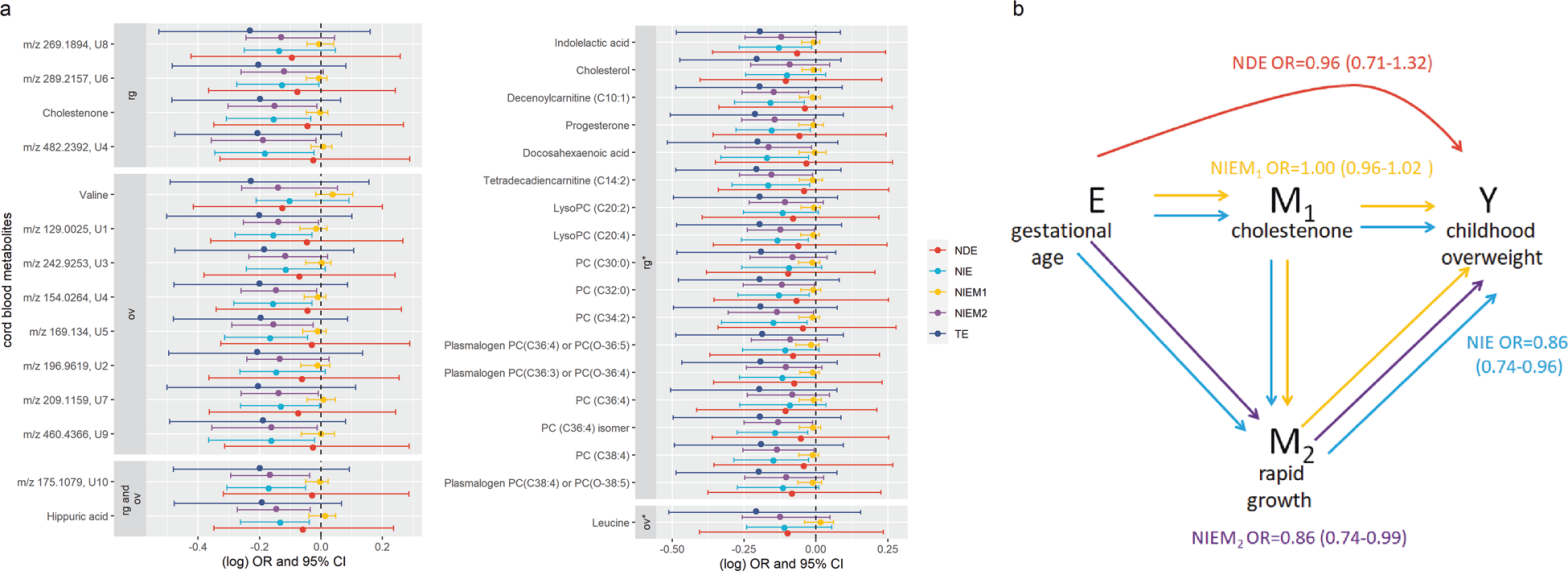
Effects of prenatal exposures on childhood overweight through cord blood metabolites and rapid postnatal growth. **a** The plot represents on the *x*-axis the (log) point estimates odds ratio (dots) and 95% confidence intervals (bars) from sequential mediation analysis for the effect of one week increase of gestation on childhood overweight through the cord blood metabolites—grouped in five sets: metabolites previously related with childhood overweight (ov), rapid growth (rg), and rapid growth and childhood overweight (rg and ov) in the MWAS, and with childhood overweight (ob*) and rapid growth (rg*) in the look-up analyses of Handakas et al. [18]—and rapid growth on the *y*-axis. The analyses are adjusted for sex of the newborns, child ethnicity, cohort membership, child age at the measurement of BMI, maternal education, pre-pregnancy BMI, smoking, age at delivery, and parity. **b** The simplified directed acyclic graph displays the causal relationships of multiple mediation for one metabolite, cholestenone, in the multiple mediation of gestation age on childhood overweight. CI: confidence intervals, NIE: natural indirect effect; NIEM_1_: natural indirect effect via M_1_; NIEM_2_: natural indirect effect via M_2_; NDE: natural direct effect; OR: odds ratio, TE: total effect.

In the multiple mediation analysis, total effects were mainly explained by the direct effect for all the exposures, with the exception of gestational age and primiparity (Fig. S5). The effect of gestational age and primiparity on childhood overweight was mainly mediated by the path involving rapid growth, with no apparent mediating role of cord blood metabolites (Fig. 4).

### Sensitivity analyses

Sensitivity analyses using IOTF cut-offs to define childhood overweight showed similar results to the main analyses (Figs. S6–8).

Using the first four principal components instead of the separate metabolites, the effect of gestational age, of primiparity and obesogenic factors score on rapid growth was partly mediated by principal component 4 (NIE OR per each week of gestation = 0.87, 95% CI = 0.81–0.93; NIE OR of primiparous vs pluriparous = 1.45, 95% CI = 1.21–1.85; NIE OR per each unit of the score = 1.11, 95% CI = 1.03–1.21; Fig. S9). Principal component 4 explained 6% of the variance of the 32 metabolic features under study and the metabolites U6 (*m/z* 289.2157), U8 (*m/z* 269.1894), tetradecadiencarnitine (C14:2) were its top contributors (with an individual contribution of >10% each) (Fig. S1). The effect of gestational age on childhood overweight was partly mediated by principal component 1 (NIE OR per each week of gestation = 1.07, 95% CI = 1.00–1.20) (Fig. S10). Principal component 1 explained 24% of the variance of the 32 metabolic features under study and three plasmalogen PCs (plasmalogen PC(C36:4) or PC(O-36:5), plasmalogen PC(C36:3) or PC(O-36:4), and plasmalogen PC(C38:4) or PC(O-38:5)) and docosahexaenoic acid, were its top contributors (with an individual contribution of >8%) (Figure S1). In the multiple mediation analysis, the effect of gestational age on childhood overweight was mediated by principal component 4 jointly with rapid growth (NIE OR per each week of gestation = 0.86, 95% CI = 0.76–0.96), but distinguishing the NIE for M_1_ and M_2_ revealed that the effect was mainly mediated by rapid growth alone (NIEM_1_ OR per each week of gestation = 1.00, 95 CI = 0.96–1.03; NIEM_2_ OR per each week of gestation = 0.86, 95 CI = 0.76–0.98, Fig. S11).

Excluding preterm births when studying the effect of gestational age, results were similar to the main analysis, except for mediation of the effect of gestational age on rapid growth by U8 (m/z 269.1894) and PC (C34:2) that were attenuated and not significant anymore, Fig. S12). Excluding children born by caesarean delivery in the single mediation analysis of prenatal exposures on rapid growth, results were similar to the main analysis, except for the mediation of the effect of maternal low vs high education on rapid growth by decenoylcarnitine (C10:1) that was attenuated and not significant anymore (Fig. S13). In addition, in this analysis, we found that the effect of the prenatal exposures examined, as summarised in the analysis of the obesogenic factors score, on rapid growth was mediated by metabolite U6 (*m/z* 289.2157, NIE OR per each unit of the score = 1.06, 95% CI = 1.01–1.15) (Fig. S13). We were not able to test mediating pathways on childhood overweight as excluding children born by caesarean delivery resulted in too small remaining sample size (*N* = 158).

## Discussion

We have investigated, for the first time, the mediating pathways leading to childhood overweight of a set of prenatal exposures (maternal education, pre-pregnancy BMI, weight gain and tobacco smoke during pregnancy, age at delivery, parity, and child gestational age) via cord blood metabolites and infant rapid growth. First, single mediation modelling, to explore if the effects of these exposures on childhood overweight were mediated by cord blood metabolites (selected as they were previously associated with rapid growth and/or childhood overweight in this study population [18]), did not show a mediating effect. Second, the effect of gestational age on childhood overweight was mainly mediated by rapid growth. Third, part of the effect of four prenatal exposures (maternal education, weight gain during the pregnancy, gestational age, and parity) on rapid growth was mediated by seven cord blood metabolites: cholestenone, decenoylcarnitine (C10:1), PC (C34:3), progesterone and three unidentified metabolites (U4, U6 and U8). Finally, using multiple mediation analysis, we investigated the sequential effect of cord blood metabolites and rapid growth in the associations between the selected prenatal exposures and childhood overweight, which confirmed the effect of gestational age on childhood overweight was mainly mediated by rapid growth while the contribution of metabolites at birth was marginal.

We found that the effect of gestational age on childhood overweight was mediated by rapid growth. A recent meta-analysis described that preterm infants have a higher risk of childhood obesity than infants born at term [14]. In preterms, growth faltering at birth induces energy conservation (thrifty) mechanisms which, in a more favourable postnatal environment (e.g., feeding with milk enriched with proteins), may result in a “thrifty catch-up fat phenotype” in infants and increased fat accumulation later in life [33]. In line with this hypothesis, our single mediation analysis showed that having a longer gestation is associated with a lower risk of being overweight and this effect is almost entirely mediated by rapid growth, suggesting rapid growth has a substantial role.

We found the effect of increasing gestation duration on decreasing risk of experiencing rapid growth was mediated by increasing levels of the metabolic feature with *m/z* 269.1894, cholestenone and PC (C34:2). The metabolic feature with *m/z* 269.1894 (U8) could not be annotated. Cholestenone is an intermediate catabolic product of conversion of cholesterol to coprostanol from bacteria within the enterohepatic circulation [34]. While coprostanol has been hypothesized to modulate cholesterol metabolism and is linked to a reduced risk of cardiovascular diseases, cholestenone’s function is poorly known [35]. PCs are essential membrane constituents and play a central role in carrying fatty acids. Previous studies showed that cord blood metabolites, including bile acids and PCs, were associated with gestational age [36] and rapid growth [18], but these associations had never been investigated in formal mediation models. In a previous study on the multi-omic signature of birthweight, we associated cholestenone and PC (C34:2) with DNA methylation at a CpG site located in the *DHCR24* gene involved in cholesterol biosynthesis suggesting that these metabolites, that we here link with gestational age, are metabolically related, and that epigenetic mechanisms may also play a role [37]. Cholestenone was the only metabolite whose mediation effect remained significant in sensitivity analyses (excluding preterm births and caesarean deliveries).

The effect of gestational age on childhood overweight was not mediated by the studied cord blood metabolites and, despite the effect of gestational age on rapid growth being mediated by three cord blood metabolites, multiple mediation models indicated that rapid growth was the main mediator of this association, while the contribution of metabolites at birth was marginal. Cord blood metabolomic studies can improve neonatal metabolic assessment beyond current knowledge through enhanced assessment of the obesogenic environment or indicating a metabolic shift that increases obesity risk [18, 38, 39]. The lack of mediation effects on childhood overweight, for the metabolites mediating the effect of gestational age on rapid growth, paves the way to different interpretations. First, cord blood metabolite concentrations were found to be transient by delivery mode [40], which could have contributed to a lack of link with childhood overweight. Second, because these cord blood metabolites mediated the effect of gestational age on rapid growth, which was inherent to their selection and rapid growth is a phenotype closer to birth, the studied metabolites may have a role in the origin of metabolic programming that we were not able to capture yet in relation to overweight later in childhood [41]. Last, the indirect effects we found in relation to rapid growth were quite small already, and they could have been further diluted in relation to childhood overweight at six years, five years later in life compared to rapid growth that was assessed at one year of age.

No clear evidence of mediation by metabolites and rapid growth on childhood overweight was found for any other prenatal exposure, apart from gestational age. However, the other prenatal exposures under study may still be involved in the propensity of childhood overweight via other *in utero* mechanisms, such as epigenetic modifications [42]. Nevertheless, we still recommend paying attention to the early prevention of childhood obesity by addressing these exposures during pregnancy.

In addition, we found that the effect of prenatal exposures (maternal education, weight gain during the pregnancy, and parity) other than gestational age on rapid growth was mediated by five cord blood metabolites: progesterone, decenoylcarnitine (C10:1), and three unidentified metabolites (U4, U6 and U8).

Progesterone levels play an important role in pregnancy, they are responsible for the implantation process and modulate the maternal immune system [43]. Maternal progesterone levels are higher at the first pregnancy [44]. Cord blood progesterone levels are higher in firstborns [45] and have been previously inversely associated with birthweight [26]. Our results showed that the risk of having a rapid growth child almost doubled in primiparae compared to multiparae, in line with previous findings [13, 46], and that progesterone mediated part of this association suggesting that progesterone levels in cord blood might be relevant for the growth of the infants. This mediation effect was also stable in sensitivity analyses considering only vaginal deliveries. A previous study showed that parity had a strong effect on cord blood metabolites, finding that the number of metabolites related to having siblings or not was more than twice that of those related to sex or delivery mode [47], but identified metabolites were mostly fatty acid metabolites, and progesterone was not among them [47].

Decenoylcarnitine (C10:1), a medium chain acylcarnitine which plays a significant role in mitochondrial energetics and fatty acid oxidation, was the only mediator specific to maternal education (not related to any other prenatal exposure under study). Lower maternal education has been previously associated with a greater risk of having an infant experiencing rapid growth [48, 49]. Maternal lower education might affect offspring weight gain indirectly through limiting access to health care, increasing exposure to stress and other detrimental factors, for example air pollution. No previous study investigated the association between child metabolomics and maternal education, however in the ALSPAC cohort children’s and adolescents’ metabolic blood profiles have been reported to differ by father’s occupation [50]. Caution in the interpretation of this finding is recommended considering that we did not find any mediating effect for the one other acylcarnitine we studied (tetradecadiencarnitine (C14:2), and that the mediation effect of decenoylcarnitine (C10:1) was lost in sensitivity analyses considering only vaginal deliveries.

Overall, the metabolites mediating the effect of prenatal exposures on rapid growth warrant further investigations because, even if they are not mediators of childhood overweight in our study, they can still be mediators of other health outcomes (e.g. cognitive outcomes). In this regard, an experimental study in rats showed cholestenone, which we found mediating the effect of gestational age on rapid growth, plays an essential role in neural stem cell differentiation [51]. Similarly, the elevated levels of progesterone, we found mediating the effect of being primiparae on rapid growth, may influence child neurodevelopment [52].

Our study’s main strength is the use of multiple mediation between factors (prenatal exposure, metabolites at birth, infant rapid growth and childhood overweight) that have a defined temporal sequence that limits reverse causality. Previous studies investigated the association between prenatal exposures, growth in infancy and obesity in childhood [8, 53, 54], but none included metabolites measured at birth. In our analysis, we considered multiple mediators in separate models. As our metabolic features were affected by a certain degree of correlation, we reduced the mediators’ dimension using PCA. We performed all the analyses using the first four principal components instead of the single metabolic features, which revealed that the effect of gestational age on childhood overweight was partly mediated by principal component 1. As we did not find the same mediation effect studying the single metabolites, metabolites are likely to have a joint mediation effect, rather than act as single identities. We measured childhood overweight based on BMI SD scores. Despite being unable to differentiate lean or fat mass, BMI is a useful measure of obesity as it correlates substantially with fat mass [55]. We used both WHO and IOTF cut-offs to define overweight, and the results were not affected by the different classifications. Furthermore, we also built a composite score, summarising each individual’s obesogenic risk due to the multiple prenatal exposures, which was strongly associated with childhood obesity and whose effect was not mediated by the metabolites or rapid growth. Finally, we also performed mediation when total effects were almost null, which allowed us to detect inconsistent mediation in case of indirect and direct effects going in opposite directions.

We acknowledge that the study has some limitations. First, the study population of the analyses had a relatively small sample size (*N* = 375 and *N* = 249 for childhood overweight and rapid growth, respectively), which translates into wide confidence intervals, and prevents subgroup analyses, for example by gender, postnatal feeding practices and delivery mode. Nevertheless, our results can still give an indication of the directionality of the effects encouraging the conduct of further observational studies [56]. Second, we acknowledge that we considered only prenatal maternal exposures while paternal behaviours and characteristics might also contribute to shaping the health of the child [57, 58]. Third, we measured metabolites in cord blood as this is easily accessible compared to adipose tissue, but metabolite composition and function may differ in the adipose tissue [59].

## Conclusion

This study provided for the first time evidence of the involvement of in utero metabolism in the propensity to rapid growth associated with maternal education, maternal weight gain, parity and gestational age. Multiple mediation analyses revealed that rapid growth explained most of the mediation effect of gestational age on childhood overweight while mediation by metabolites was marginal. Further observational studies are still required to support the results and provide more precise estimates.

## Supplementary information

Supplementary Material

## Data Availability

The metabolites used in the analyses can be found in the EXPOsOMICS metabolome dataset available via the MetaboLights repository with the Accession no. MTBLS1684.

## Code availability

Code relevant to the analyses is available upon request to the corresponding author.
