# Supplementary material for: Cord blood metabolites and rapid postnatal growth as multiple mediators in the prenatal propensity to childhood overweight
Source: Int J Obes (Lond). 2022 May 4;46(7):1384–93. doi: 10.1038/s41366-022-01108-0 (PMC9239910; doi:10.1038/s41366-022-01108-0)
Supplement: Supplementary file 1 — Supplementary Material [file 41366_2022_1108_MOESM1_ESM.pdf]

Supporting information to

Cord blood metabolites and rapid postnatal growth as multiple mediators in the prenatal propensity to childhood overweight

Rossella Alfano[1,2]†*, Michelle Plusquin[1]†, Oliver Robinson[2], Sonia Brescianini[3], Lida Chatzi[4], Pekka Keski-Rahkonen[5], Evangelos Handakas[2], Lea Maitre[6], Tim Nawrot[1], Nivonirina Robinot[5], Theano Roumeliotaki[7], Franco Sassi[8], Augustin Scalbert[5], Martine Vrijheid[6,9,10], Paolo Vineis[2], Lorenzo Richiardi[11], Daniela Zugna[11]

[1]Centre for Environmental Sciences, Hasselt University, Diepenbeek, Belgium

[2]Medical Research Council-Health Protection Agency Centre for Environment and Health, Imperial College London, London, United Kingdom

[3] Centre for Behavioural Science and Mental Health, Istituto Superiore di Sanità, Rome, Italy

[4]Department of Preventive Medicine, Keck School of Medicine, University of Southern California, Los Angeles, United States of America

[5]Nutrition and Metabolism Branch, International Agency for Research on Cancer, Lyon, France

[6]Barcelona Institute of Global Health (ISGlobal), Barcelona, Spain

[7]Department of Social Medicine, Faculty of Medicine, University of Crete, Heraklion, Greece

[8]Centre for Health Economics & Policy Innovation, Department of Economics & Public Policy, Imperial College Business School, South Kensington Campus, London, UK

[9]Universitat Pompeu Fabra (UPF), Barcelona, Spain

[10]CIBER Epidemiología y Salud Pública (CIBERESP), Spain

[11]Cancer Epidemiology Unit, Department of Medical Sciences, University of Turin and CPO-Piemonte, Torino, Italy

† These authors have contributed equally to this work and share first authorship

Table of Content

Supplementary Method 4

Table S1 Descriptive characteristics of the study population participating to the analyses. 5

Table S2 Score assigned to each prenatal factor concurring to the obesogenic factors score calculation. 6

Table S3 List of the 31 metabolites from the study of Handakas et al.. Handakas E, Keski-Rahkonen P, Chatzi L, et al. Cord blood metabolic signatures predictive of childhood overweight and rapid growth. Int J Obes. 2021. doi:10.1038/s41366-021-00888-1. 8

Table S4 Association with rapid growth and childhood overweight upon adjustment for confounders. 10

Figure S1 Heatmap showing Pearson correlation coefficients and scree plot from principal components analyses of the 32 cord blood metabolic features under study in the population participating to (**a**) the single mediation analysis of the effect of prenatal exposures on postnatal rapid growth mediated by metabolites (N=375) and (**b**) to all the analyses on childhood overweight (N=249). 11

Figure S2 Results of the single mediation analysis of the effect of prenatal exposures on infancy rapid growth through the cord blood metabolites (N=375). 12

Figure S3 Results of the single mediation analysis of the effect of (**a**) maternal education, (**b**) maternal weight gain during the pregnancy, (**c**) gestational age, and (**d**) parity on the postnatal rapid growth through the metabolites on the postnatal rapid growth through the metabolites in the subset of 249 children participating to the analyses of childhood overweight. 13

Figure S4 Results of the single mediation analysis of the effect of prenatal exposures on childhood overweight through the cord blood metabolites (N=245). 14

Figure S5 Results of sequential multiple mediation of prenatal exposures on childhood overweight through the cord blood metabolites and postnatal rapid growth (N=245). 15

Figure S6 Results of the single mediation analysis of the effect of prenatal exposures on childhood overweight defined using IOTF cut-offs through the cord blood metabolites (N=245). 16

Figure S7 Results of the single mediation analysis of the effect of prenatal exposures on childhood overweight defined using IOTF cut-offs through postnatal rapid growth (N=245). . 17

Figure S8 Results of sequential mediation of prenatal on childhood overweight defined using IOTF cut-offs through the cord blood metabolites and postnatal rapid growth (N=245). 18

Figure S9 Results of the single mediation analysis of the effect of prenatal exposures on childhood overweight through the principal components (PCs) of the cord blood metabolites. 19

Figure S10 Results of the single mediation analysis of the effect of prenatal exposures on rapid postnatal growth through the two principal components (PCs) of the cord blood metabolites. . 20

Figure S11 Results of sequential mediation of prenatal on childhood overweight through the principal components (PCs) of cord blood metabolites and rapid postnatal growth. 21

Figure S12 Results from sensitivity analyses excluding the preterm birth of (**a**) single mediation of gestational age on postnatal rapid growth through cord blood metabolites and (**b**) on childhood overweight through rapid growth, (**c**) on childhood overweight through cord blood metabolites,

and (**d**) sequential mediation of gestational age on childhood overweight through cord blood metabolites and postnatal rapid growth. 22

Figure S13 Results from sensitivity analyses excluding children born by caesarean delivery of the effect of prenatal exposures on infancy rapid growth through the cord blood metabolites (N=276). 23

Supplementary Methods

Cohort specific study population

ENVIR*ON*AGE is an ongoing population-based prospective birth cohort study that aims at exploring the human ageing and its interaction with the environment [1]. From February 2010 onward, more than 2,000 mother-infant pairs have been recruited at delivery at the East-Limburg Hospital in Genk (Belgium). The study was approved by the ethics committee of the Hasselt University and the Hospital East-Limburg, Genk, Belgium and conducted in accordance with the Declaration of Helsinki. Anthropometric data were self-reported by the parents until the age of 2 years, and measured by trained-staff during a follow-up sub-study when the child was two years of age.

INMA is a population-based birth cohort including 3,768 mother-child pairs enrolled between 1997 and 2008 during the first prenatal visits at different health care centres Spain [2]. The study was approved by the ethics committee of the Hospital del Mar Medical Research Institute and conducted according to principles of the Helsinki Declaration. This study is based on 657 mother-child pairs recruited at two health care centres in Sabadell. Anthropometric data were collected via surveys or measured until at least adolescence.

Piccolipiù is a birth cohort of more than 3,000 mother-child pairs recruited in five Italian cities (Turin, Trieste, Viareggio, Firenze and Rome) [3]. Ethical approvals have been obtained from the Ethics committees of the Local Health Unit Roma E (management centre), of the Istituto Superiore di Sanità (National Institute of Public Health) and of each local centre. This study is based on 544 mother-child pairs recruited at the Sant'Anna Hospital in Turin. Follow-up anthropometric data collection surveys occurred at 6, 12 and 24 months after the delivery and then when the children turned 4 and 6 years with direct measurements at a clinical visit.

Rhea is a prospective mother-child cohort of 1,610 mother-child pairs recruited between 2007 and 2008 during the first trimester pregnancy visits at the public primary health care centres or hospitals in Heraklion, Greece [4, 5]. The Ethics Committee of the University Hospital at Heraklion approved the study protocols. Follow-up anthropometric measurements were collected via surveys or measured for the participants are available up to 11 years.

Informed consent for participation to each study was provided by parents or mothers.

References

1. Janssen BG, Madhloum N, Gyselaers W, et al. Cohort Profile: The ENVIRonmental influence ON early AGEing (ENVIRONAGE): a birth cohort study. Int J Epidemiol. 2017;46(5):1386-7m. doi:10.1093/ije/dyw269

2. Guxens M, Ballester F, Espada M, et al. Cohort Profile: the INMA--INfancia y Medio Ambiente--(Environment and Childhood) Project. Int J Epidemiol. 2012;41(4):930-40. doi:10.1093/ije/dyr054

3. Farchi S, Forastiere F, Vecchi Brumatti L, et al. Piccolipiu, a multicenter birth cohort in Italy: protocol of the study. BMC Pediatr. 2014;14:36. doi:10.1186/1471-2431-14-36

4. Chatzi L, Plana E, Daraki V, et al. Metabolic syndrome in early pregnancy and risk of preterm birth. Am J Epidemiol. 2009;170(7):829-36. doi:10.1093/aje/kwp211

5. Chatzi L, Leventakou V, Vafeiadi M, et al. Cohort Profile: The Mother-Child Cohort in Crete, Greece (Rhea Study). Int J Epidemiol. 2017;46(5):1392-3k. doi:10.1093/ije/dyx084

Table S1 Descriptive characteristics of the study population participating to the analyses.

	**Study population rapid growth analyses** N= 375	**Study population childhood overweight** N=249
Cohort membership,		
ENVIRONAGE	104 (27.73)	
INMA	86 (22.93)	83 (33.33)
Piccolipiù	97 (25.87)	78 (31.33)
Rhea	88 (23.47)	88 (35.34)
Sex, boy	191 (50.93)	127 (51.00)
Child ethnicity, maternal origin different from study country	33 (8.80)	14 (5.62)
Delivery, caesarean	99 (26.47)	91 (36.69)
Gestation age, weeks	39.20 (1.57)	39.23 (1.54)
Gestation age, <37 weeks	25 (6.67)	14 (5.62)
Parity, nulliparous	177 (47.20)	104 (41.77)
Maternal age, years	31.03 (4.52)	31.79 (4.74)
Maternal age, >=35years	220 (58.67)	127 (51.00)
Maternal education level,		
(i) Low (no diploma or primary school diploma),	42 (11.20)	29 (11.65)
(ii) Medium (secondary school diploma),	158 (42.13)	120 (48.19)
(iii) High (university degree or higher education qualification)	175 (46.67)	100 (40.16)
Pre-pregnancy maternal BMI, Kilograms/ meters^2	23.82 (4.52)	23.72 (4.56)
Pre-pregnancy maternal BMI,		
(i) Normal (BMI<25)	263 (70.13)	172 (69.08)
(ii) Overweight or obese (BMI≥25)	112 (29.87)	77 (30.92)
Maternal weight gain over the pregnancy, Kilograms	13.55 (5.18)	13.24 (5.09)
Maternal weight gain over the pregnancy, excess	121 (32.27)	76 (30.52)
Maternal pregnancy tobacco smoke, yes	65 (17.33)	49 (19.68)
Obesogenic factors score, numeric (range 0-7)	2.24 (1.32)	2.15 (1.34)
Postnatal rapid growth at 12 months, yes	107 (28.53)	70 (28.11)
Child overweight (WHO cut-off), yes	56 (21.96)	56 (22.49)
Child overweight (IOTF cut-offs), yes	62 (24.31)	60 (24.10)
Child age at the BMI measurement, years	5.37 (1.01)	5.40 (1.00)

Number (%) and mean (±standard deviation) are reported for categorical and continuous variables, respectively. IOTF: International Obesity Task Force; BMI: body mass index; WHO: World Health Organization

Table S2 Scores we assigned to each prenatal factor concurring to the obesogenic factors score calculation. The scores were based on a-priori knowledge, and for each prenatal exposure we reported at least one reference reporting an association with childhood overweight and obesity.

**Prenatal exposure**	**score**
maternal education[1],	
university degree or higher education	0
secondary school diploma	0.5
no diploma or primary school diploma	1
pre-pregnancy maternal BMI[2,3],	
<25	0
>=25	1
maternal weight gain during the pregnancy[3],	
<=18 (for underweight mothers)<br>*<=16 (for normal weight mothers)*<br>*<=11.5 (for overweight mothers)*<br>*<=9 (for obese mothers)*	0
>18 (for underweight mothers)<br>*>16 (for normal weight mothers)*<br>*>11.5 (for overweight mothers)*<br>*>9 (for obese mothers)*	1
maternal tobacco smoke[4],	
no	0
yes	1
maternal age[5],	
>=32	0
<32	1
gestational age[6],	
>=37	0
<37	1
Parity[7],	
multiparous	0
nulliparous	1

0: low risk according to a-priori knowledge; 1: high risk according to a-priori knowledge

References

1. Lamerz A, Kuepper-Nybelen J, Wehle C, Bruning N, Trost-Brinkhues G, Brenner H, Hebebrand J, Herpertz-Dahlmann B. Social class, parental education, and obesity prevalence in a study of six-year-old children in Germany. *Int J Obes* (Lond) 2005;29(4):373-80. doi: 10.1038/sj.ijo.0802914 [published Online First: 2005/03/16]
2. Heslehurst N, Vieira R, Akhter Z, et al. The association between maternal body mass index and child obesity: A systematic review and meta-analysis. *PLoS Med.* 2019;16(6):e1002817.
3. Voerman E, Santos S, Patro Golab B, et al. Maternal body mass index, gestational weight gain, and the risk of overweight and obesity across childhood: An individual participant data meta-analysis. *PLoS Med.* 2019;16(2):e1002744.
4. von Kries R, Toschke AM, Koletzko B, Slikker W, Jr. Maternal Smoking during Pregnancy and Childhood Obesity. *American Journal of Epidemiology.* 2002;156(10):954-961.

5. Liu S, Lei J, Ma J, et al. Interaction between delivery mode and maternal age in predicting overweight and obesity in 1,123 Chinese preschool children. *Ann Transl Med.* 2020;8(7):474-474.
6. Ou-Yang MC, Sun Y, Liebowitz M, et al. Accelerated weight gain, prematurity, and the risk of childhood obesity: A meta-analysis and systematic review. *PLoS One.* 2020;15(5):e0232238.
7. Gaillard R, Rurangirwa AA, Williams MA, et al. Maternal parity, fetal and childhood growth, and cardiometabolic risk factors. *Hypertension.* 2014;64(2):266-274.

Table S3 List of the 31 metabolites from the study of Handakas et al.. Handakas E, Keski-Rahkonen P, Chatzi L, et al. Cord blood metabolic signatures predictive of childhood overweight and rapid growth. Int J Obes. 2021. doi:10.1038/s41366-021-00888-1.

N	*m/z*	Rt(min)	Compound name	Level of identification confidence[a]
Metabolites associated in the MWAS with postnatal rapid growth[b]				
1	269.1894	5.31	Unidentified (U8)	4
2	289.2157	4.83	Unidentified (U6)	4
3	385.3487	9.08	Cholestenone	1
4	482.2392	3.66	Unidentified (U4)[c]	4
Metabolites associated in the MWAS with childhood overweight[b]				
5	72.08108	0.80	Valine	1
6	129.0025	0.49	Unidentified (U1)	4
7	242.9253	0.58	Unidentified (U3)	4
4	154.0264	0.69	Unidentified (U4)[c]	4
8	169.1340	0.70	Unidentified (U5)	4
9	196.9619	0.53	Unidentified (U2)	4
10	209.1159	6.17	Unidentified (U7)	4
11	460.4366	8.54	Unidentified (U9)	4
Metabolites associated in the MWAS with 12 months postnatal rapid growth and childhood overweight[d]				
12	175.1079	0.68	Unidentified (10)	4
13	202.0483	3.07	Hippuric acid	1
Metabolites associated in the look-up analyses with 12 months postnatal rapid growth[b]				
14	206.0822	3.83	Indolelactic acid	1
15	369.3521	9.06	Cholesterol	1
16	314.2321	4.88	Decenoylcarnitine (C10:1)	2
17	315.2320	6.39	Progesterone	1
18	329.2482	7.23	Docosahexaenoic acid	1
19	368.2793	5.63	Tetradecadiencarnitine (C14:2)	2
3	385.3487	9.08	Cholestenone	1
20	548.3681	7.14	LysoPC (C20:2)	2
21	563.3141	6.89	LysoPC (C20:4)	2
22	706.5410	8.49	PC (C30:0)	2
23	734.5700	8.96	PC (C32:0)	2
24	758.5747	8.68	PC (C34:2)	2
25	766.5815	8.86	Plasmalogen PC(C36:4) or PC(O-36:5)	2
26	768.5883	9.19	Plasmalogen PC(C36:3) or PC(O-36:4)	2
27	782.5722	9.06	PC (C36:4)	2
28	793.5614	8.63	PC (C36:4) isomer	2
29	810.6053	9.17	PC (C38:4)	2
30	794.6046	9.78	Plasmalogen PC(C38:4) or PC(O-38:5)	2
Metabolites associated in the look-up analyses with childhood overweight[b]				
31	132.1021	1.45	Leucine	1
18	329.2482	7.23	Docosahexaenoic acid	1

m/z: mass to charge ratio; Rt: retention time; min: minutes.

[a]As proposed by Metabolomics Standards Initiative (Sumner LW, Amberg A, Barrett D, et al. Proposed minimum reporting standards for chemical analysis Chemical Analysis Working Group (CAWG) Metabolomics Standards Initiative (MSI). Metabolomics. 2007;3(3):211-21. doi:10.1007/s11306-007-0082-2):

Level 1 (identified compounds): retention time and MS/MS spectra matches with an authentic chemical standard;

Level 2 (putatively annotated compound): no standard available or analysed but a database candidate within 5 ppm mass error, plausible retention time, matching isotope pattern and MS/MS spectra;

Level 3 (putatively characterized compound class): MS/MS spectral similarity with compounds from a known chemical class and plausible retention time;

Level 4 (unidentified compounds): unidentiable compound

[b]false discovery rate (FDR) p value for the association <0.05

[c]please note that the same metabolite was found associated with postnatal rapid growth and childhood overweight. However, two different metabolic features had the highest intensity in the two analyses and hence both were added in the present study

[d]p value for the association <0.05

Metabolites included in multiple analyses are coulored in red

Table S4 Association with rapid growth and childhood overweight upon adjustment for confounders.

	**Rapid growth OR (95%CI)**	**Childhood overweight OR (95%CI)**
Maternal education level, *Low vs high*	1.85 (0.83-4.03)	1.21 (0.42-3.36)
Pre-pregnancy maternal BMI, Kilograms/ meters^2	1.01 (0.96-1.07)	**1.10 (1.02-1.18)**
Maternal weight gain over the pregnancy, Kilograms	0.98 (0.94-1.03)	1.04 (0.97-1.11)
Maternal pregnancy tobacco smoke, yes	1.30 (0.69-2.43)	1.51 (0.66-3.36)
Maternal age, years	1.01 (0.95-1.07)	0.98 (0.90-1.06)
Gestation age, weeks	**0.56 (0.46-0.67)**	0.80 (0.62-1.02)
Parity, primiparous	**1.93 (1.14-1.31)**	1.90 (0.91-4.01)
Obesogenic factors score, numeric (range 0-7)	**1.33 (1.11-1.61)**	**1.61 (1.25-2.11)**
Rapid growth	**-**	**4.39 (2.14-9.28)**

All the analyses are adjusted for sex of the newborns, child ethnicity, cohort membership and child age (for childhood overweight analyses only). The analyses of maternal pre-pregnancy BMI, maternal weight gain, smoking, age at delivery, gestational age and parity are additionally adjusted for maternal education, pre-pregnancy BMI, smoking, age at delivery, and parity.

Figure S1 Heatmaps showing Pearson correlation coefficients, scree plots, loading plots and plots showing first 10 contributors to the first and fourth principal components from principal components analyses of the 32 cord blood metabolic features under study in the population participating to (**a**) the single mediation analysis of the effect of prenatal exposures on postnatal rapid growth mediated by metabolites (N=375) and (**b**) to all the analyses on childhood overweight (N=249).

a

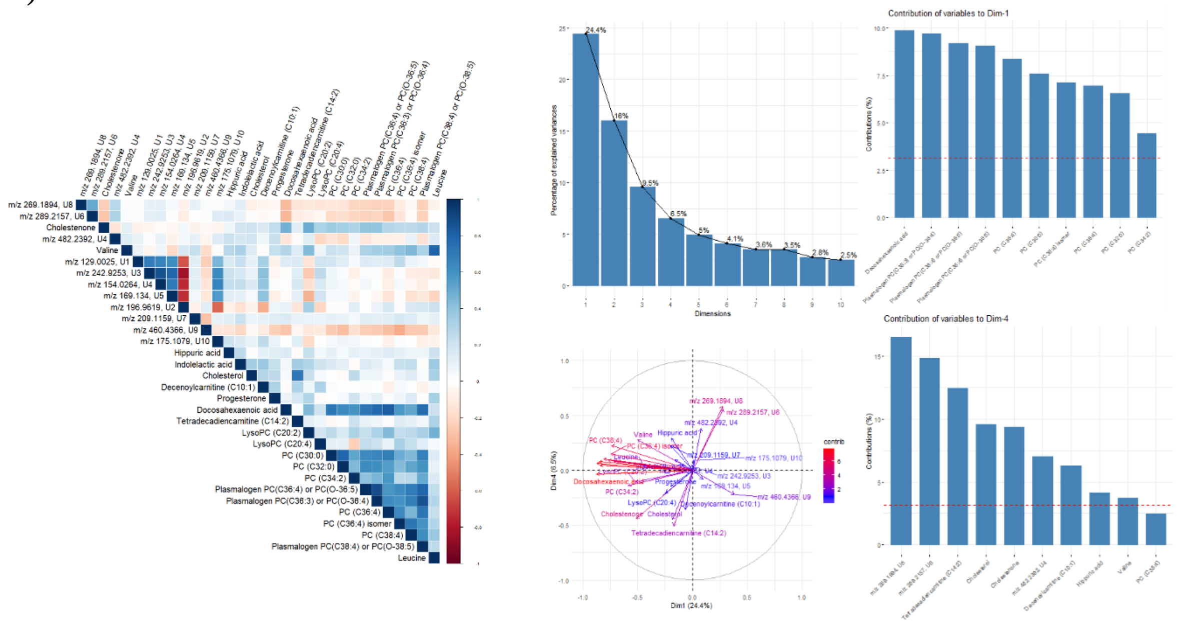

b

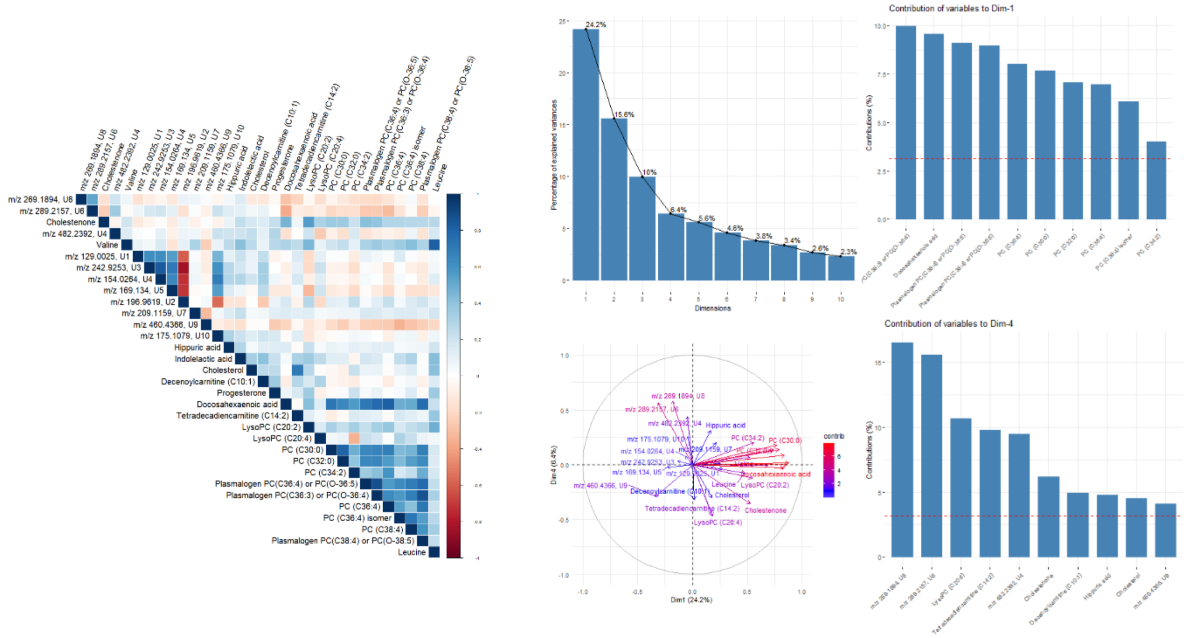

Figure S2 Results of the single mediation analysis of the effect of prenatal exposures on infancy rapid growth through the cord blood metabolites (N=375).

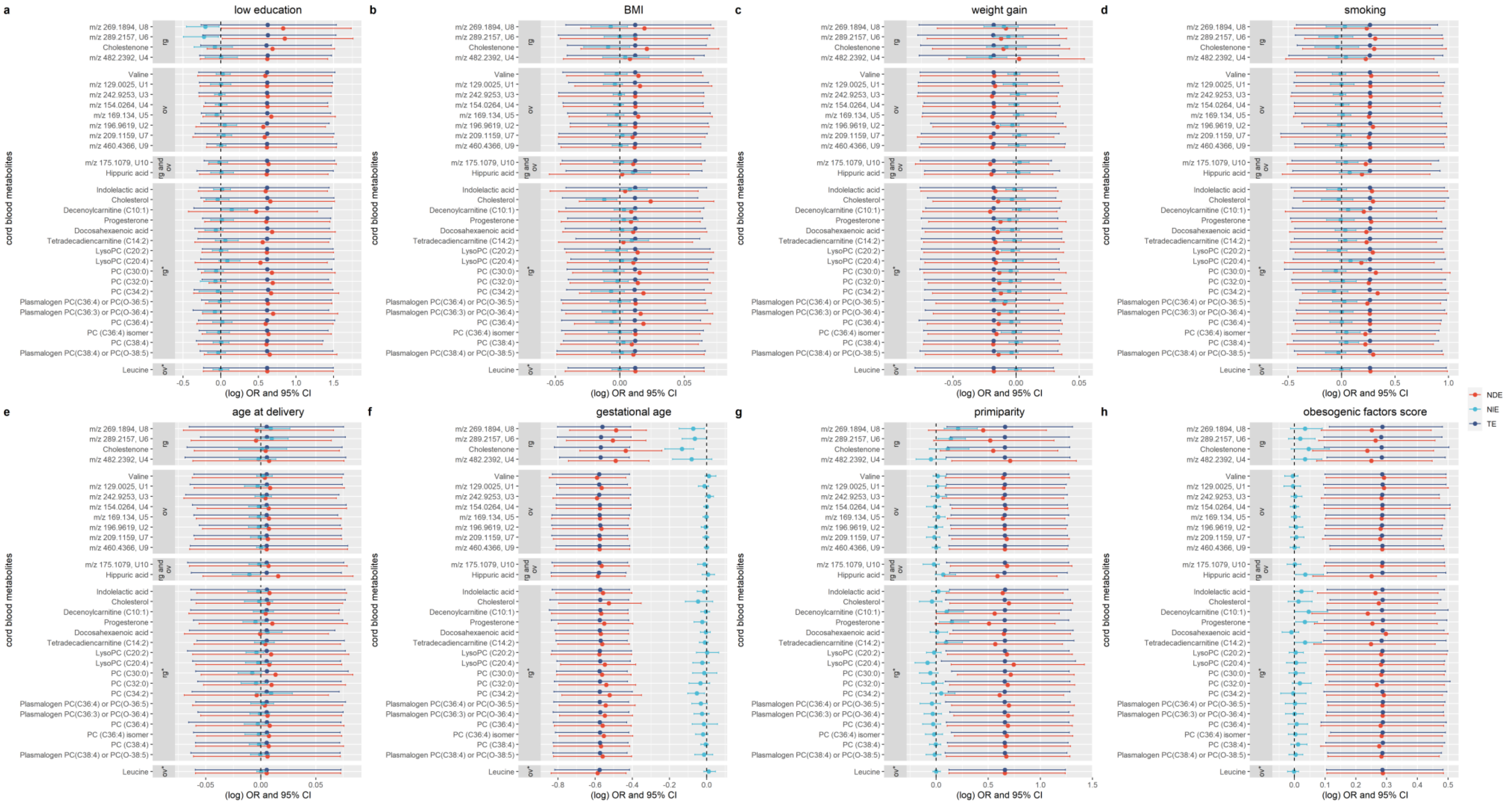

CI: confidence intervals; NIE: natural indirect effect; NDE: natural direct effect; OR: odds ratio; TE: total effect

Metabolites are grouped in five sets: metabolites previously related with childhood overweight (ob), rapid growth (rg), and rapid growth and childhood overweight (rg and ov) in the MWAS and with childhood overweight (ob*), rapid growth (rg*) in the look-up analyses of Handakras et al.. Handakas E, Keski-Rahkonen P, Chatzi L, et al. Cord blood metabolic signatures predictive of childhood overweight and rapid growth. Int J Obes. 2021. doi:10.1038/s41366-021-00888-1

Figure S3 Results of the single mediation analysis of the effect of (**a**) maternal education, (**b**) maternal weight gain during the pregnancy, (**c**) gestational age, and (**d**) parity on the postnatal rapid growth through the metabolites on the postnatal rapid growth through the metabolites in the subset of 249 children participating to the analyses of childhood overweight.

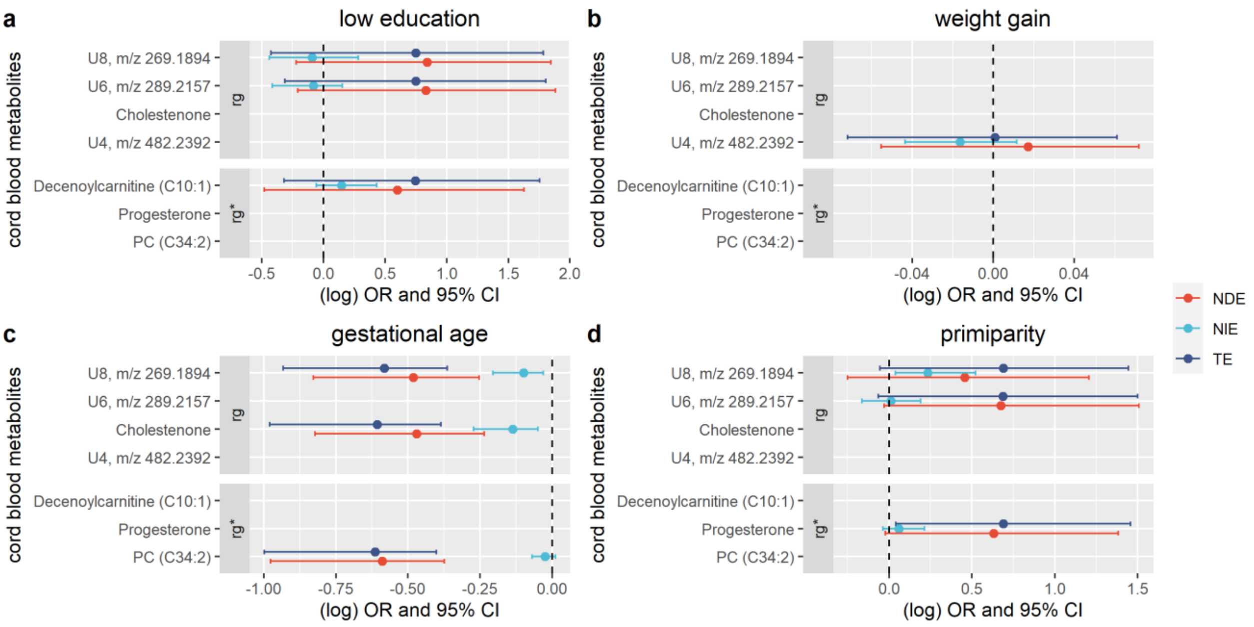

CI: confidence intervals; NIE: natural indirect effect; NDE: natural direct effect; OR: odds ratio; TE: total effect

Metabolites are grouped in two sets: metabolites previously related rapid growth in the MWAS (rg) and in the look-up analyses (rg*) of Handakras et al.. Handakas E, Keski-Rahkonen P, Chatzi L, et al. Cord blood metabolic signatures predictive of childhood overweight and rapid growth. Int J Obes. 2021. doi:10.1038/s41366-021-00888-1

Figure S4 Results of the single mediation analysis of the effect of prenatal exposures on childhood overweight through the cord blood metabolites (N=245).

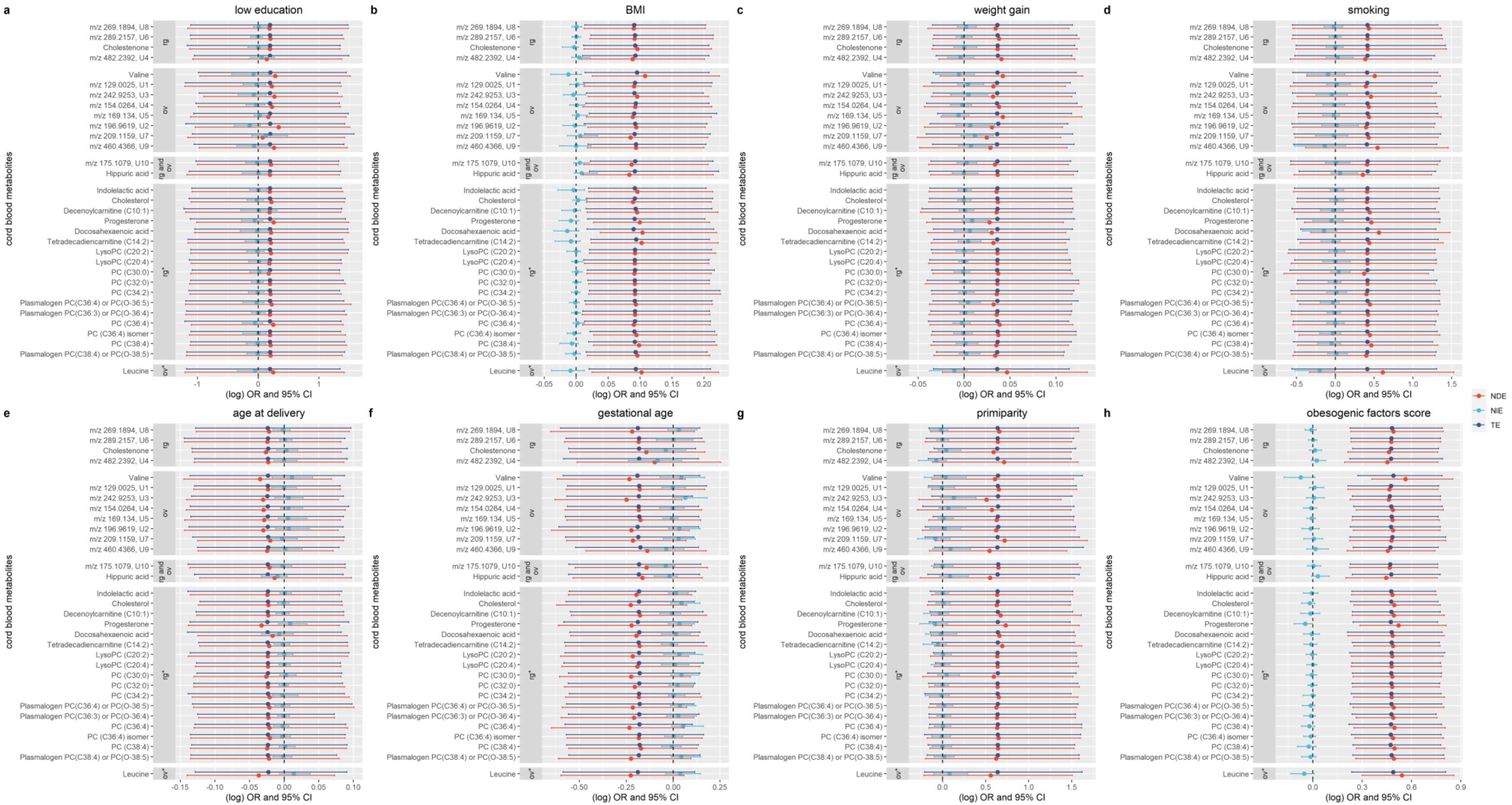

CI: confidence intervals; NIE: natural indirect effect; NDE: natural direct effect; OR: odds ratio; TE: total effect

Metabolites are grouped in five sets: metabolites previously related with childhood overweight (ob), rapid growth (rg), and rapid growth and childhood overweight (rg and ov) in the MWAS and with childhood overweight (ob*), rapid growth (rg*) in the look-up analyses of Handakras et al.. Handakas E, Keski-Rahkonen P, Chatzi L, et al. Cord blood metabolic signatures predictive of childhood overweight and rapid growth. Int J Obes. 2021. doi:10.1038/s41366-021-00888-1

Figure S5 Results of sequential multiple mediation of prenatal exposures on childhood overweight through the cord blood metabolites and postnatal rapid growth (N=245).

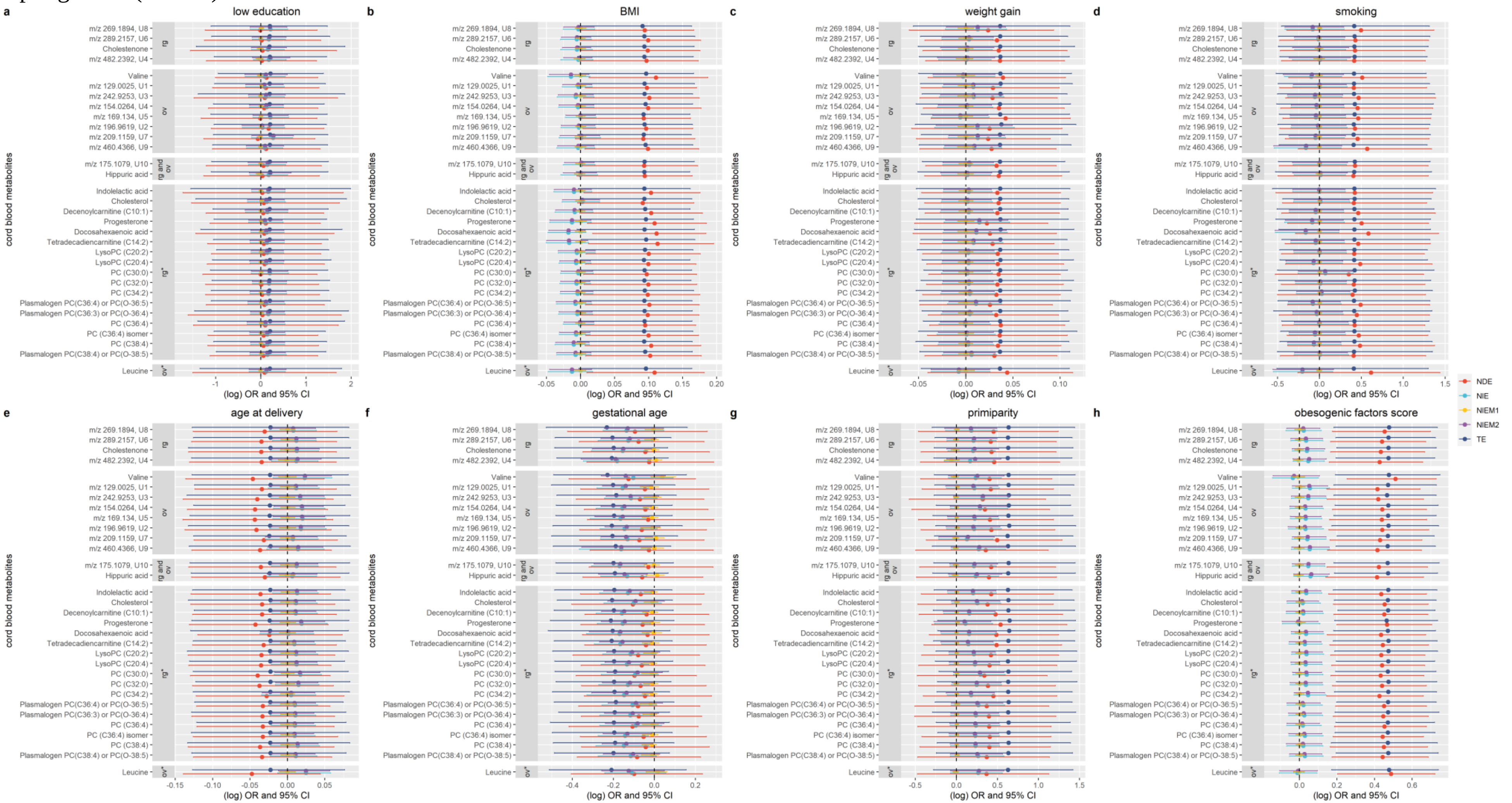

CI: confidence intervals; NIE: natural indirect effect; NIEM$_1$/M$_2$: natural indirect effect via M$_1$/M$_2$; NDE: natural direct effect; OR: odds ratio; TE: total effect
Metabolites are grouped in five sets: metabolites previously related with childhood overweight (ob), rapid growth (rg), and rapid growth and childhood overweight (rg and ov) in the MWAS and with childhood overweight (ob*), rapid growth (rg*) in the look-up analyses of Handakras et al.. Handakas E, Keski-Rahkonen P, Chatzi L, et al. Cord blood metabolic signatures predictive of childhood overweight and rapid growth. Int J Obes. 2021. doi:10.1038/s41366-021-00888-1

Figure S6 Results of the single mediation analysis of the effect of prenatal exposures on childhood overweight defined using IOTF cut-offs through the cord blood metabolites (N=245).

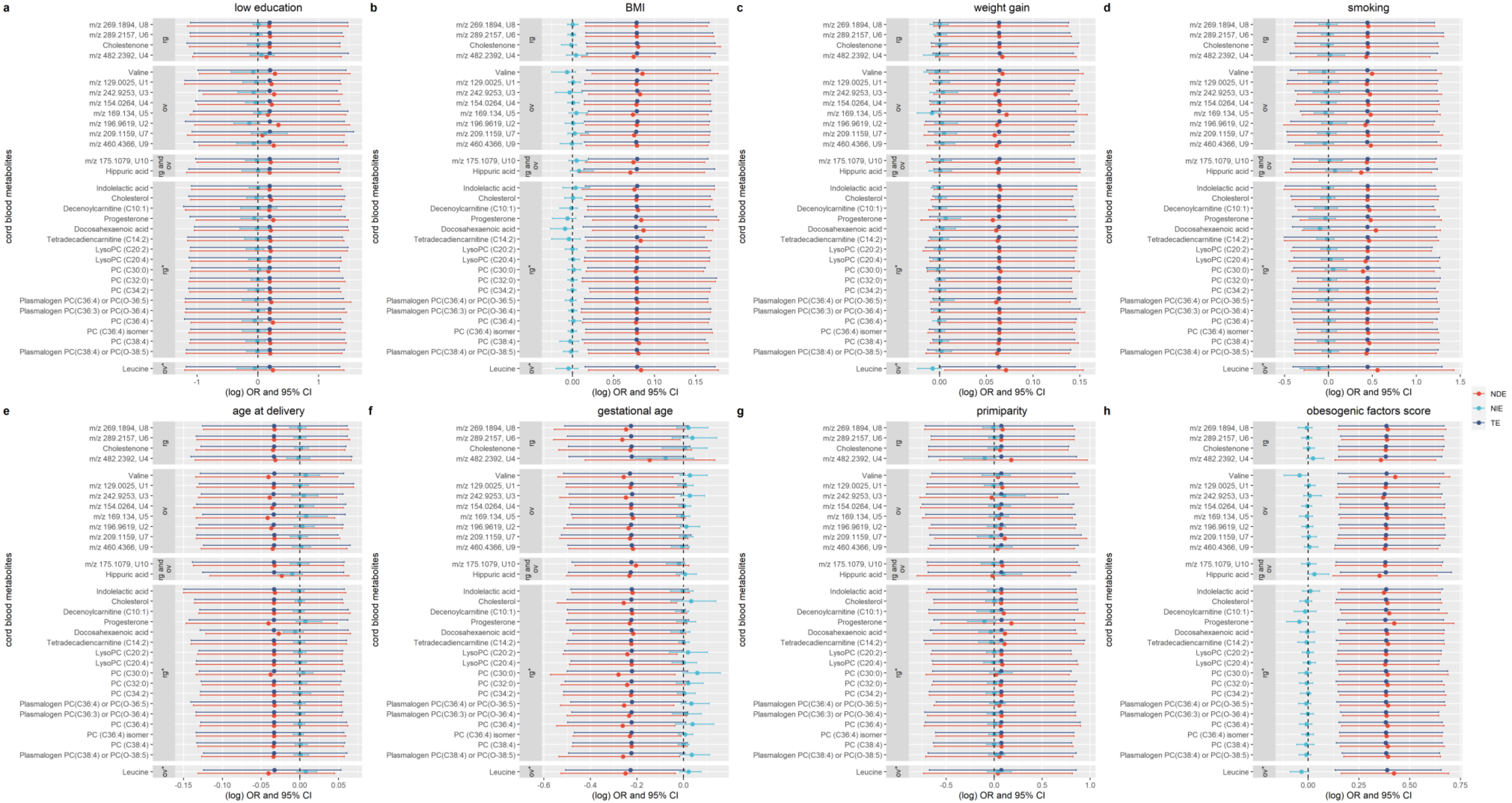

CI: confidence intervals; NIE: natural indirect effect; NDE: natural direct effect; OR: odds ratio; TE: total effect

Metabolites are grouped in five sets: metabolites previously related with childhood overweight (ob), rapid growth (rg), and rapid growth and childhood overweight (rg and ov) in the MWAS and with childhood overweight (ob*), rapid growth (rg*) in the look-up analyses of Handakras et al.. Handakas E, Keski-Rahkonen P, Chatzi L, et al. Cord blood metabolic signatures predictive of childhood overweight and rapid growth. Int J Obes. 2021. doi:10.1038/s41366-021-00888-1

Figure S7 Results of the single mediation analysis of the effect of prenatal exposures on childhood overweight defined using IOTF cut-offs through postnatal rapid growth (N=245).

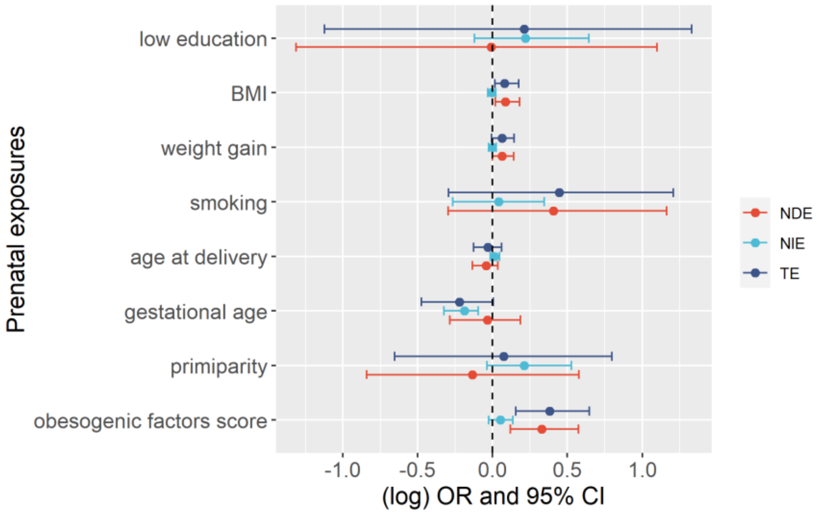

CI: confidence intervals; NIE: natural indirect effect; NDE: natural direct effect; OR: odds ratio; TE: total effect

Figure S8 Results of sequential mediation of prenatal on childhood overweight defined using IOTF cut-offs through the cord blood metabolites and postnatal rapid growth (N=245).

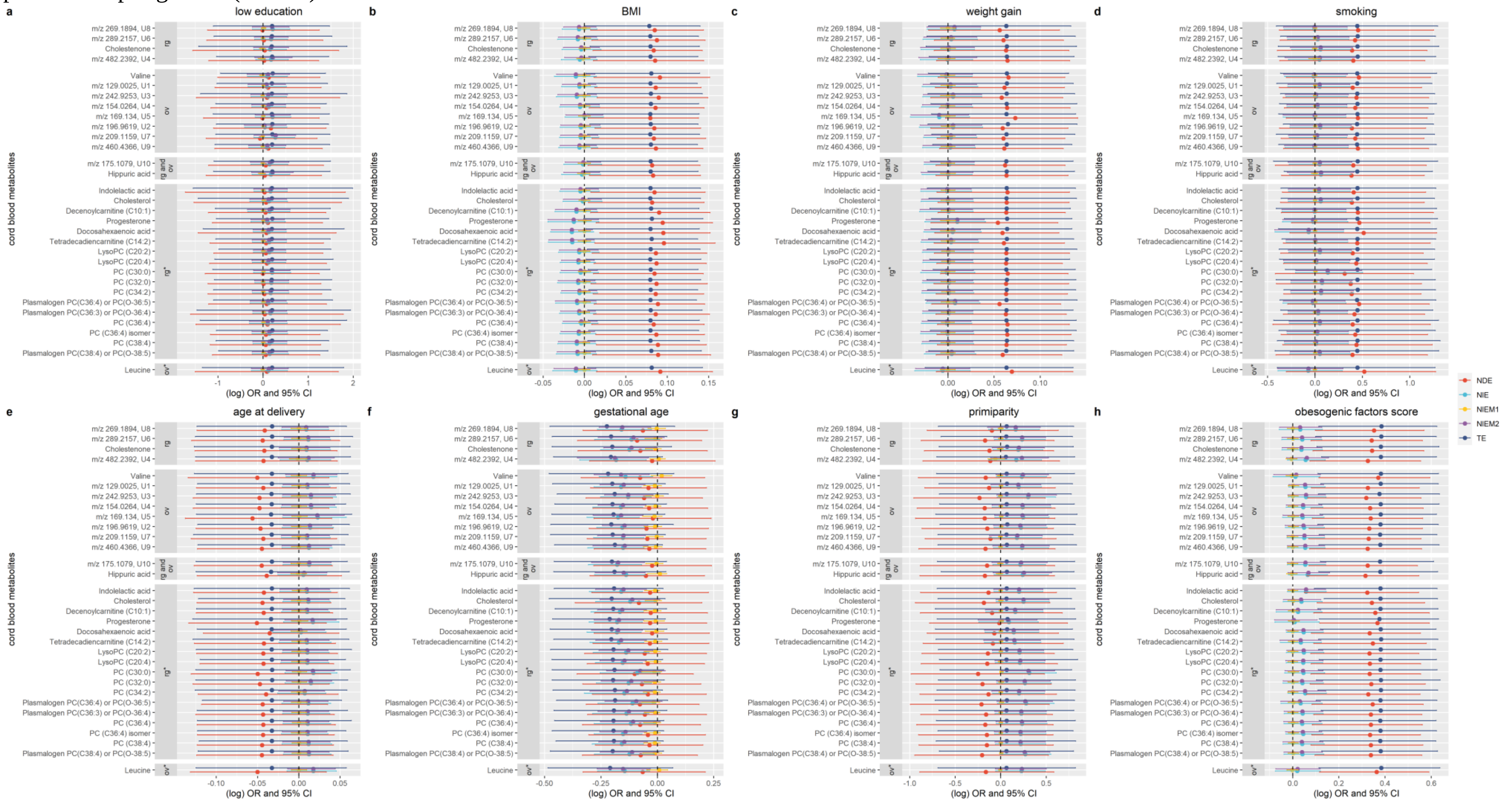

CI: confidence intervals; NIE: natural indirect effect; NIEM$_1$/M$_2$: natural indirect effect via M$_1$/M$_2$; NDE: natural direct effect; OR: odds ratio; TE: total effect
Metabolites are grouped in five sets: metabolites previously related with childhood overweight (ob), rapid growth (rg), and rapid growth and childhood overweight (rg and ov) in the MWAS and with childhood overweight (ob*), rapid growth (rg*) in the look-up analyses of Handakras et al.. Handakas E, Keski-Rahkonen P, Chatzi L, et al. Cord blood metabolic signatures predictive of childhood overweight and rapid growth. Int J Obes. 2021. doi:10.1038/s41366-021-00888-1

Figure S9 Results of the single mediation analysis of the effect of prenatal exposures on rapid postnatal growth through the principal components (PCs) of the cord blood metabolites.

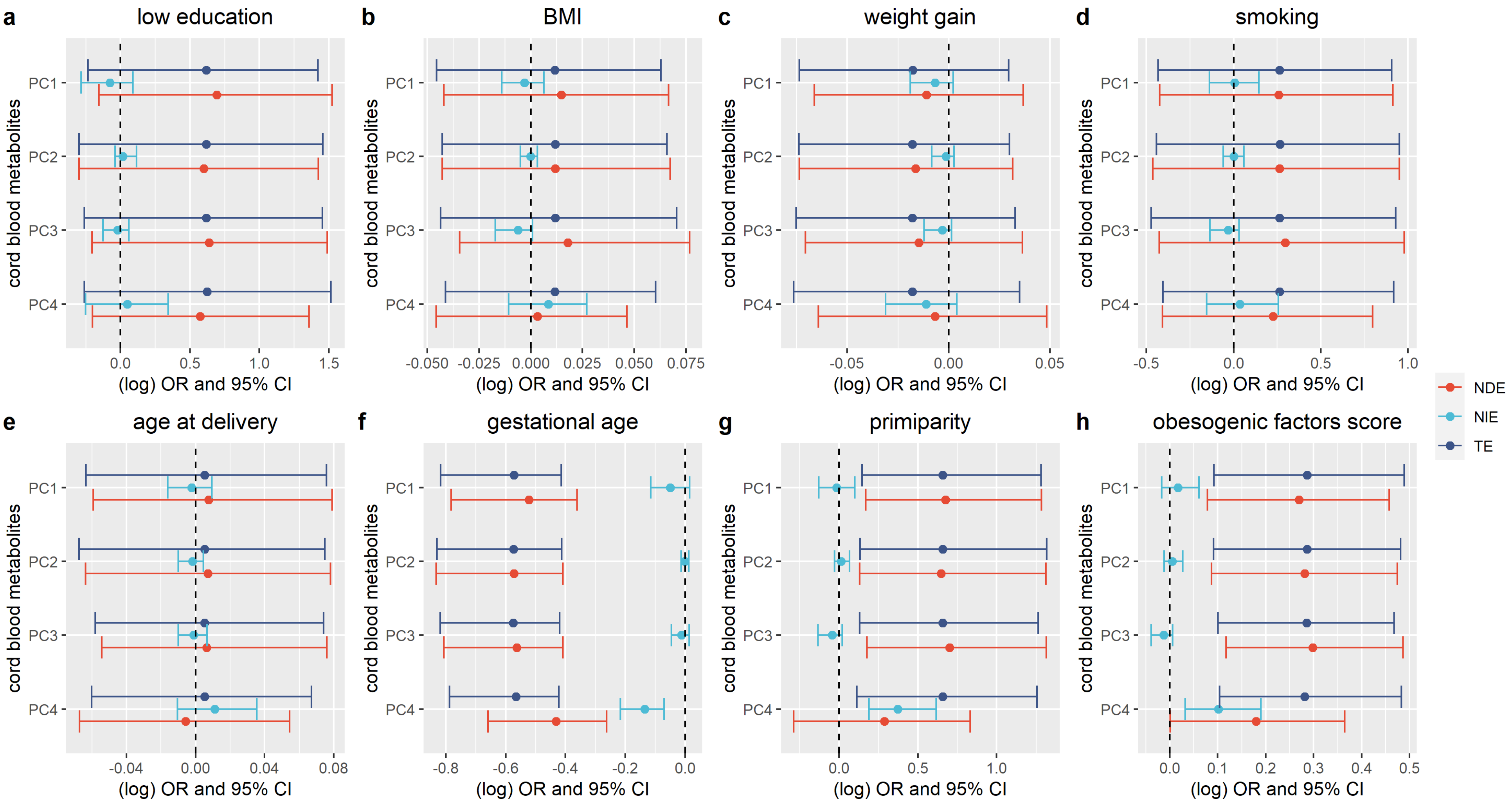

CI: confidence intervals; NIE: natural indirect effect; NDE: natural direct effect; OR: odds ratio; PC: principal component; TE: total effect

Figure S10 Results of the single mediation analysis of the effect of prenatal exposures on childhood overweight through the two principal components (PCs) of the cord blood metabolites.

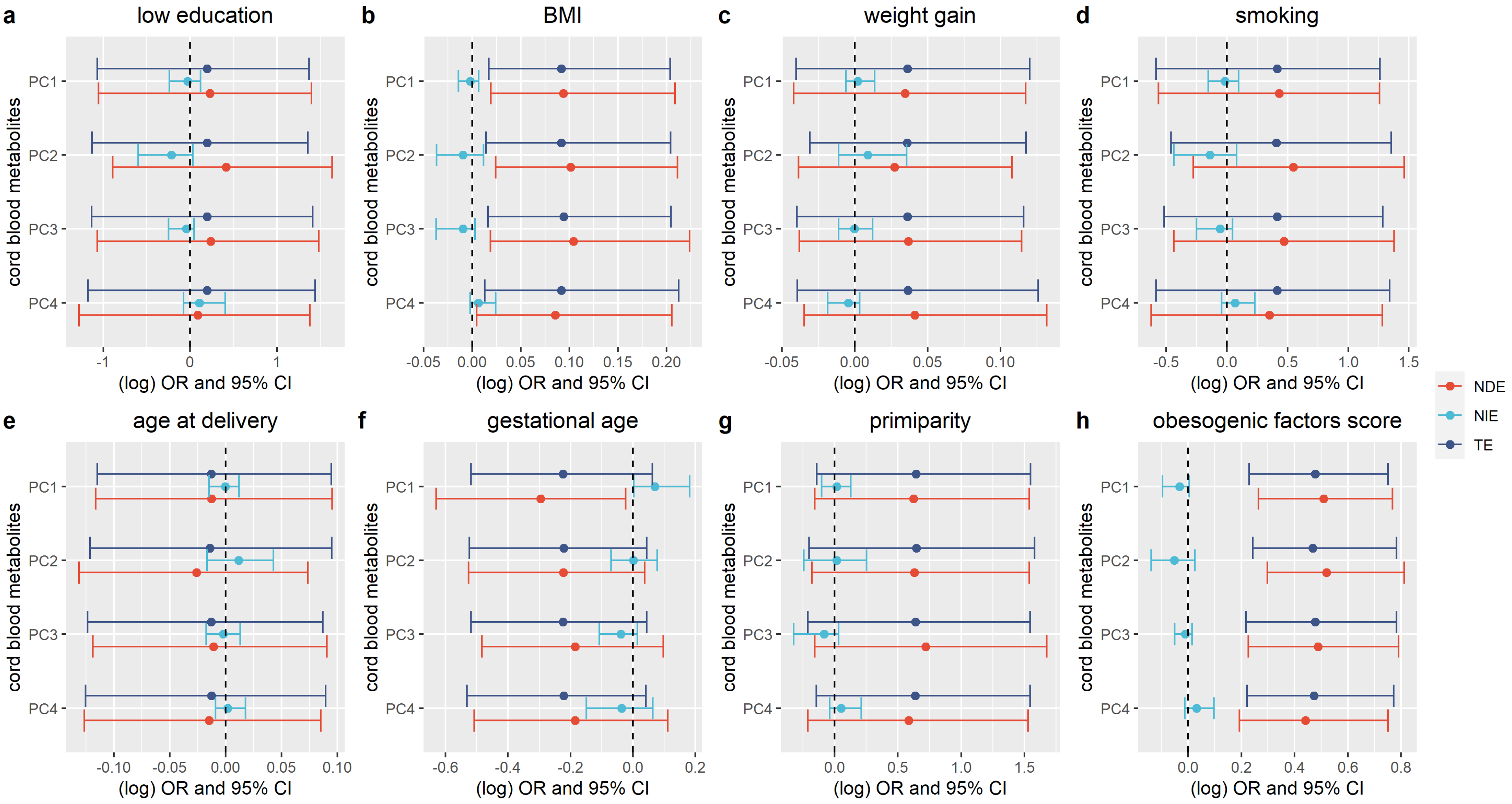

CI: confidence intervals; NIE: natural indirect effect; NDE: natural direct effect; OR: odds ratio; PC: principal component; TE: total effect

Figure S11 Results of sequential mediation of prenatal on childhood overweight through the principal components (PCs) of cord blood metabolites and rapid postnatal growth.

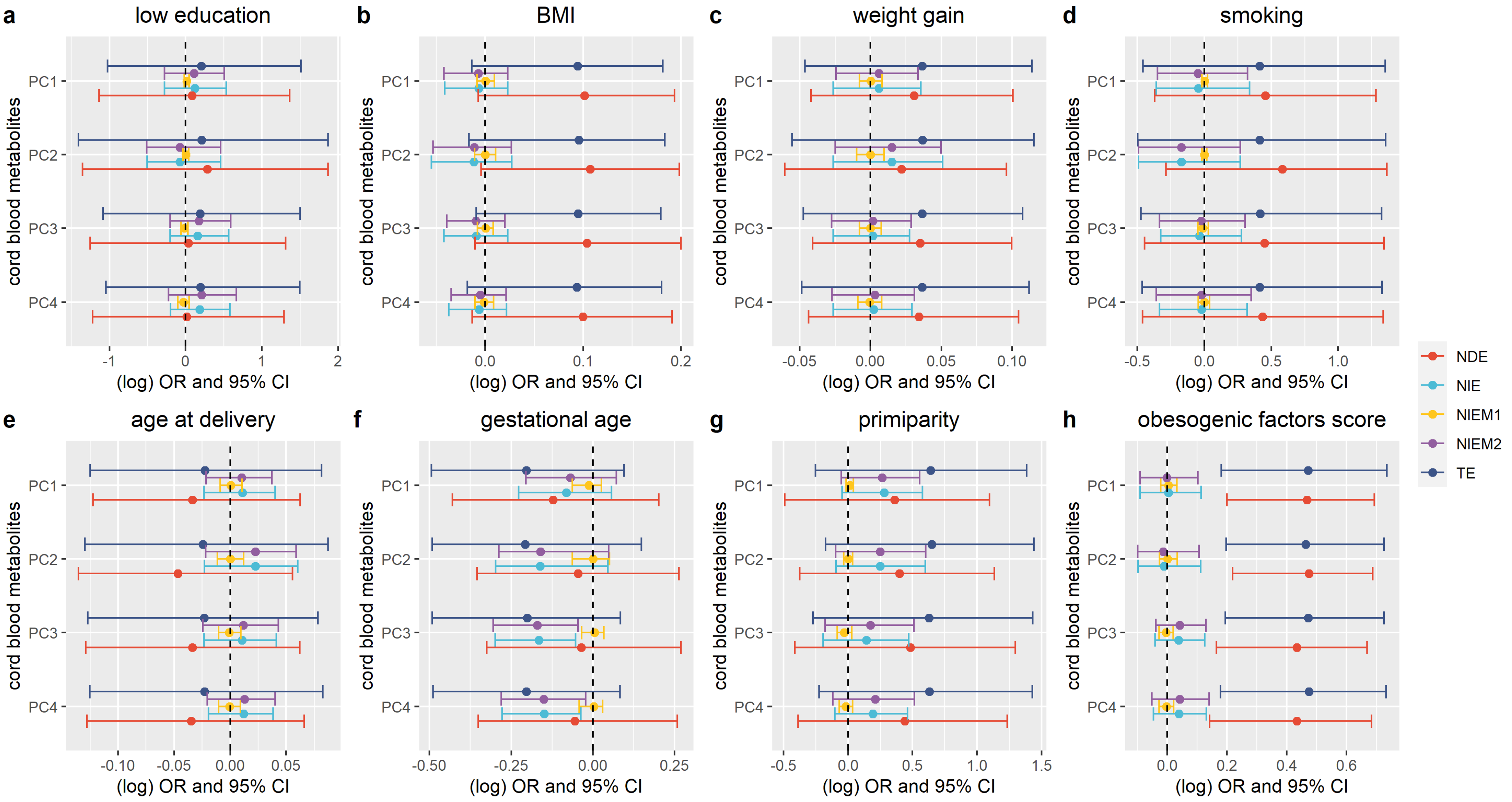

CI: confidence intervals; NIE: natural indirect effect; NIEM$_1$/M$_2$: natural indirect effect via M$_1$/M$_2$; NDE: natural direct effect; OR: odds ratio, PC: principal component; TE: total effect

Figure S12 Results from sensitivity analyses excluding the preterm birth of (**a**) single mediation of gestational age on postnatal rapid growth through cord blood metabolites and (**b**) on childhood overweight through rapid growth, (**c**) on childhood overweight through cord blood metabolites, and (**d**) sequential mediation of gestational age on childhood overweight through cord blood metabolites and postnatal rapid growth.

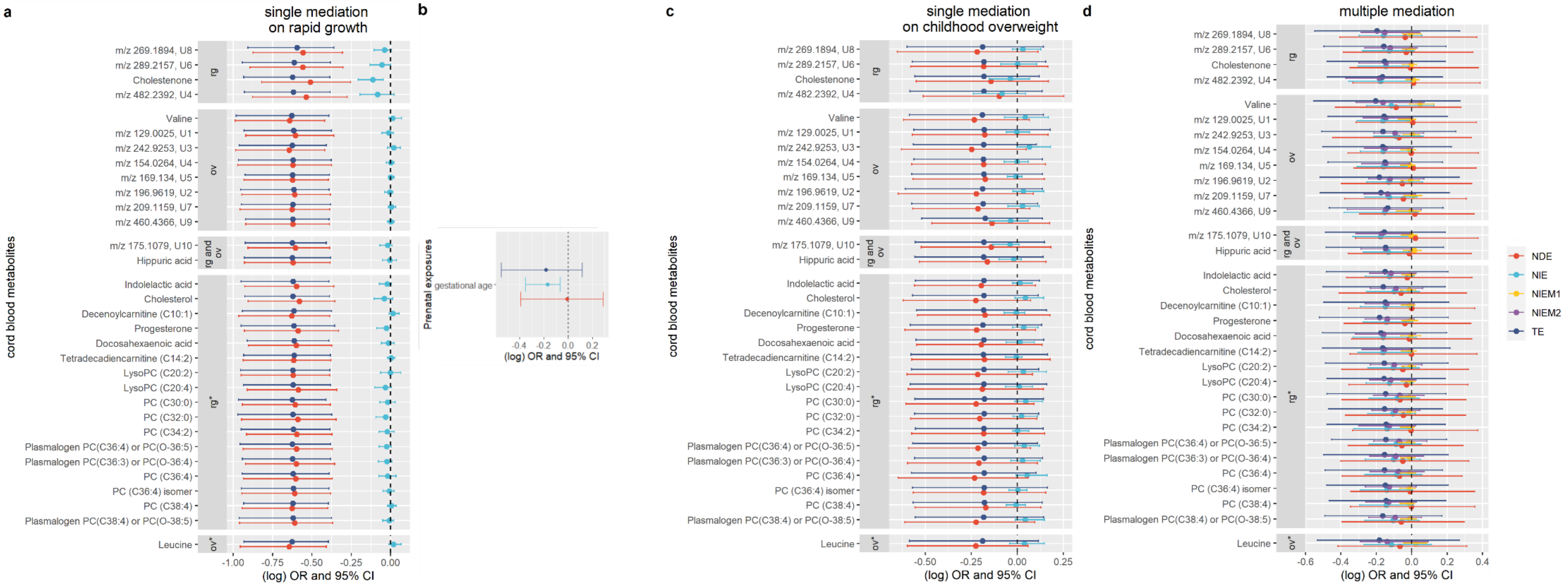

CI: confidence intervals, NIE: natural indirect effect; $NIEM_1$: natural indirect effect via M_1; $NIEM_2$: natural indirect effect via M_2; NDE: natural direct effect; OR: odds ratio; TE: total effect

Excluding the preterm births, results were similar to main analysis: the effect of gestational age on rapid growth was mediated by cholestenone (NIE OR per each week of gestation= 0.89, 95% CI= 0.81-0.95, TE OR per each week of gestation= 0.54 per each week of gestation, 95% CI= 0.39-0.70) while mediation by U8 (m/z 269.1894) and PC (C34:2) attenuated and were not significant anymore; the effect of gestational age on childhood overweight was mediated by rapid growth (NIE OR per each week of gestation= 0.84, 95% CI= 0.71-0.94, TE OR per each week of gestation=0.83 per each week of gestation, 95% CI=0.59-1.16); no metabolite mediated the effect of any studied exposure on childhood overweight and the multiple mediation confirmed the effect of gestational age on childhood overweight was mostly mediated by rapid growth

Figure S13 Results from sensitivity analyses excluding children born by caesarean delivery of the effect of prenatal exposures on infancy rapid growth through the cord blood metabolites (N=276).

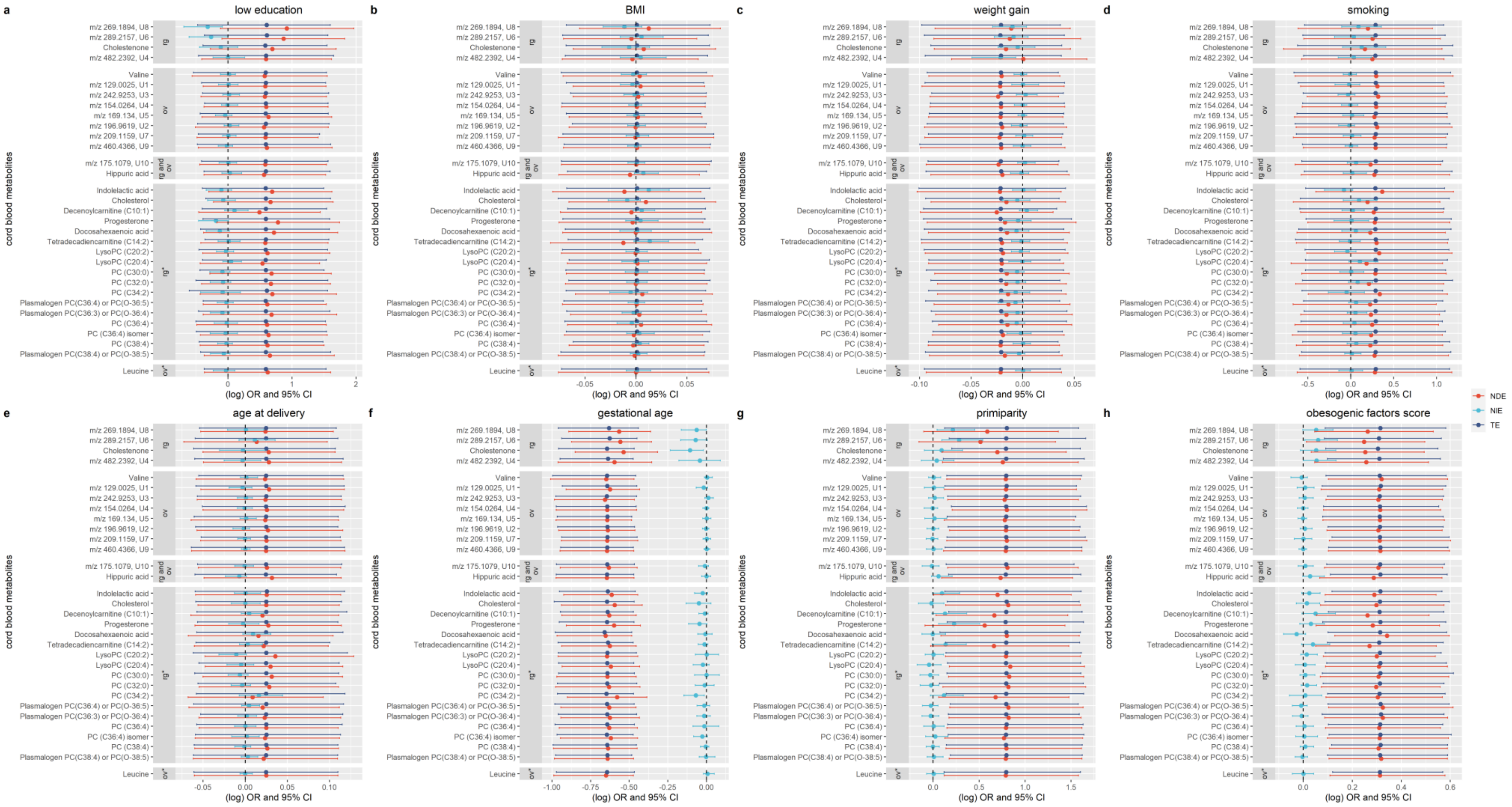

Excluding children born by caesarean delivery, results were similar to main analysis: the effect of maternal low vs high education on rapid growth was mediated by metabolite U6 (m/z 289.2157, NIE OR of maternal low vs high education= 0.77, 95% CI= 0.55-1.00) and metabolite U8 (m/z 269.1894,

NIE OR of maternal low vs high education= 0.73, 95% CI= 0.50-0.91) while mediation by decenoylcarnitine (C10:1) attenuated and was not significant anymore; the effect of gestational age on rapid growth was mediated by cholestenone (NIE OR per each week of gestation= 0.90, 95% CI= 0.79-0.98), U8 (m/z 269.1894, NIE OR per each week of gestation= 0.94, 95% CI= 0.85-1.00) and PC (C34:2) (NIE OR per each week of gestation= 0.93, 95% CI= 0.86-0.99); the effect of being primiparous on rapid growth was mediated by metabolite U6 (m/z 289.2157, NIE OR of primiparous vs pluriparous= 1.33, 95% CI= 1.10-1.72), metabolite U8 (m/z 269.1894, NIE OR of primiparous vs pluriparous= 1.24, 95% CI= 1.04-1.57) and progesterone (NIE OR of primiparous vs pluriparous= 1.25, 95% CI= 1.06-1.66) (Figure S13). In addition, we found that the effect of the prenatal exposures examined, as summarized in the analysis of the obesogenic factors score, on rapid growth was mediated by metabolite U6 (m/z 289.2157, NIE OR per each unit of the score= 1.06, 95% CI= 1.01-1.15). We were not able to test mediating pathways on childhood overweight since excluding children born by caesarean delivery resulted in too small remaining sample size (N=158).
